# A role of stochastic phenotype switching in generating mosaic endothelial cell heterogeneity

**DOI:** 10.1038/ncomms10160

**Published:** 2016-01-08

**Authors:** Lei Yuan, Gary C. Chan, David Beeler, Lauren Janes, Katherine C. Spokes, Harita Dharaneeswaran, Anahita Mojiri, William J. Adams, Tracey Sciuto, Guillermo Garcia-Cardeña, Grietje Molema, Peter M. Kang, Nadia Jahroudi, Philip A. Marsden, Ann Dvorak, Erzsébet Ravasz Regan, William C. Aird

**Affiliations:** 1 Center for Vascular Biology Research, Department of Medicine, Beth Israel Deaconess Medical Center, Boston, Massachusetts 02215, USA; 2 Department of Medicine, Beth Israel Deaconess Medical Center, Boston, Massachusetts 02215, USA; 3 Department of Medicine, University of Alberta, Edmonton, Alberta T6G 2R3, Canada; 4 Department of Pathology, Beth Israel Deaconess Medical Center, Boston, Massachusetts 02215, USA; 5 Center for Excellence in Vascular Biology, Department of Pathology, Brigham and Women's Hospital, Boston, Massachusetts 02215, USA; 6 Department of Pathology and Medical Biology, Medical Biology Section, University Medical Center Groningen, University of Groningen, 9700 AB Groningen, The Netherlands; 7 Cardiovascular Institute, Department of Medicine, Beth Israel Deaconess Medical Center, Boston, Massachusetts 02215, USA; 8 Department of Medicine, University of Toronto, Toronto, Ontario M5G 2C4, Canada; 9 St. Michaels's Hospital, Toronto, Ontario M5B 1W8, Canada

## Abstract

Previous studies have shown that biological noise may drive dynamic phenotypic mosaicism in isogenic unicellular organisms. However, there is no evidence for a similar mechanism operating in metazoans. Here we show that the endothelial-restricted gene, von Willebrand factor (*VWF*), is expressed in a mosaic pattern in the capillaries of many vascular beds and in the aorta. In capillaries, the mosaicism is dynamically regulated, with VWF switching between ON and OFF states during the lifetime of the animal. Clonal analysis of cultured endothelial cells reveals that dynamic mosaic heterogeneity is controlled by a low-barrier, noise-sensitive bistable switch that involves random transitions in the DNA methylation status of the *VWF* promoter. Finally, the hearts of VWF-null mice demonstrate an abnormal endothelial phenotype as well as cardiac dysfunction. Together, these findings suggest a novel stochastic phenotype switching strategy for adaptive homoeostasis in the adult vasculature.

## Abstract (web summary)

Spontaneous phenotypic heterogeneity confers a population-level advantage to cells that are exposed to fluctuating environments. Here the authors show that the endothelium of some organs displays a dynamic mosaicism in expression of Von Willebrand factor, suggesting bet hedging as a strategy for adaptive homeostasis.

## Graphical abstract

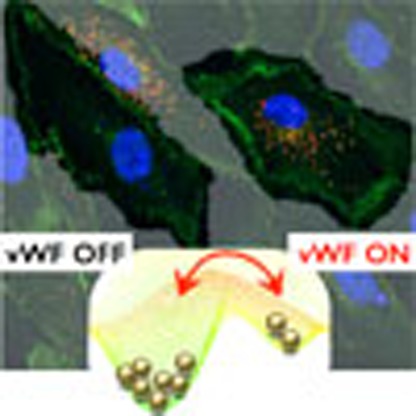

Endothelial cells from veins and arteries and from capillaries of different organs demonstrate heterogeneity at structural, functional and molecular levels^1^,^2^. Intriguingly, phenotypic heterogeneity also exists between neighbouring endothelial cells exposed to the same extracellular environment^3^,^4^. For example, we, and others, have shown that von Willebrand factor (VWF), a circulating glycoprotein that mediates platelet adhesion to the subendothelial surface of injured blood vessels^5^, is expressed in a mosaic pattern in the aorta and in selected capillary beds^6^,^7^,^8^,^9^.

It is widely held that phenotypic heterogeneity is driven by deterministic mechanisms, including hardwired cellular responses to the extracellular environment and/or epigenetic memory^3^,^10^. However, recent evidence points to a role for biological noise in generating mosaic expression among isogenic cells^11^,^12^,^13^. Noise is all-pervasive in cells, arising from omnipresent random fluctuations in the normal kinetics of molecular processes such as transcription and translation^14^,^15^,^16^,^17^,^18^,^19^,^20^. Noise typically results in a normal ‘spread' in gene and protein expression around the mean, as illustrated on the quasi-energy landscape in Fig. 1a (refs 4, 11, 21, 22). In multistable regulatory systems, however, noise can drive transitions between distinct, locally stable states (Fig. 1b), converting a graded response to a binary response^4^,^23^. Previous studies in prokaryotes and single-cell eukaryotes have implicated an adaptive role for biological noise in bet hedging and in task sharing/allocation^24^,^25^,^26^,^27^,^28^,^29^. In higher eukaryotes, noise has been shown to trigger random cell fate decisions during development^23^,^30^,^31^,^32^,^33^,^34^,^35^,^36^. However, to date, there is no evidence that stochastic phenotype switching occurs in differentiated cells within mammals, or that it plays a role in adult homoeostasis.

In this study, we hypothesized that VWF mosaicism is driven by dynamic noise-induced transitions between ON and OFF VWF states. To test this hypothesis, we developed genetically modified mouse models that allow for a comparison of VWF expression in a single snapshot in time with its cumulative expression over time (Fig. 1c,d). We show that VWF flickers ON and OFF in the endothelium of some, but not all vascular beds. Using *in vitro* cell culture, we demonstrate that dynamic VWF mosaicism is generated by the ON/OFF toggle of a low-barrier, noise-sensitive bistable switch, and that it involves random transitions in *VWF* promoter DNA methylation. Finally, we present data that supports a role for mosaicism in endothelial health. Collectively, these findings suggest that biological noise is exploited by selected vascular beds to generate adaptive neighborhood-level phenotypic diversity, providing new insights into mechanisms of endothelial cell heterogeneity.

## Results

### VWF is expressed in a mosaic pattern *in vivo*

Previous studies have shown that VWF expression in the endothelium is higher in veins compared with arteries and in large vessels compared with capillaries^37^,^38^. In immunofluorescent co-localization studies of VWF and the pan-endothelial marker CD31, we found that VWF expression is also heterogeneous at the level of single vessels. For example, VWF protein was detected in a minority of endothelial cells in the capillaries of adult heart, skeletal muscle, lung and brain (Fig. 2a shows heart). Consistent with previous reports^6^,^7^, the aorta also consisted of micro-domains of VWF-positive and -negative endothelial cells (Fig. 2b). In contrast to these mosaic patterns, VWF expression was ubiquitous in venous endothelium (Fig. 2c shows heart), and virtually undetectable in kidney glomeruli and liver sinusoids (Fig. 2d shows liver). We previously targeted the *lacZ* reporter gene to the endogenous mouse *Vwf* locus (Supplementary Fig. 1a), and showed that expression of the reporter gene in heterozygous mice (vWF^LacZ/+^) mirrored that of the endogenous gene^9^. Indeed, LacZ expression followed a similarly mosaic pattern in the capillaries of heart, skeletal muscle, lung and brain, as well as in the aorta (Fig. 2a,b; Supplementary Fig. 2). Together, these findings indicate that at any point in time, VWF expression varies not only between organs and blood vessel types, but also between neighbouring endothelial cells.

### Mosaic VWF expression is dynamic in selected vascular beds

**Figure 1:**
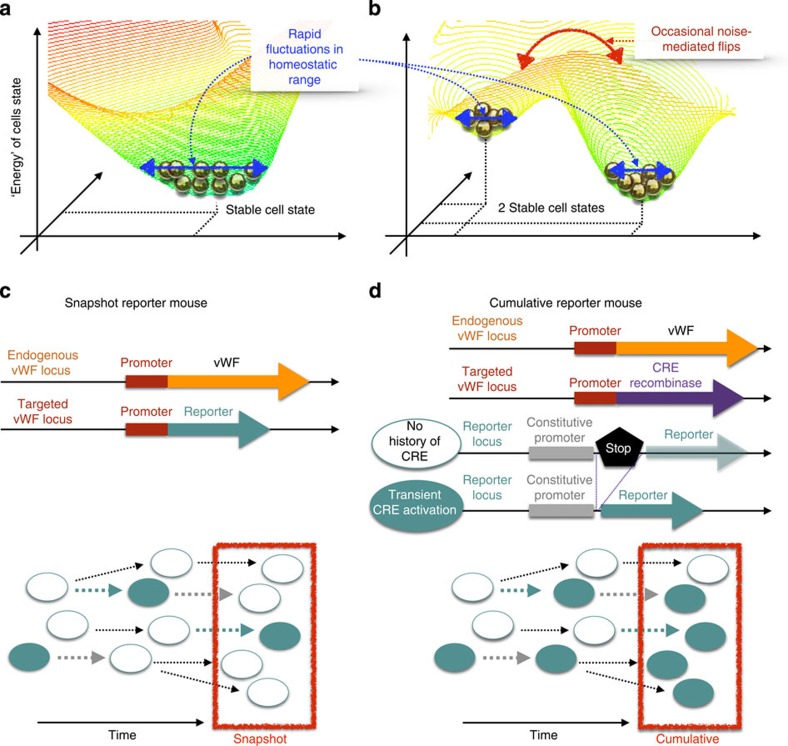
Schematics of noise-sensitive bistable regulatory circuits and mouse models. (**a**) Schematic (quasi)-energy landscape of a homoeostatic regulatory circuit with one stable state, where biological noise generates a continuous spread of slightly different cell states around the lowest-energy stable state (double-headed blue arrow). (**b**) Schematic (quasi)-energy landscape of a bistable regulatory circuit with two stable states, where biological noise not only generates continuous spreads around each stable state (double-headed blue arrows), but can occasionally lead to abrupt, discrete transitions between the two distinct states (double-headed red arrow). (**c**) Top, Schematic representation of the gene targeting strategy for creating a mouse a model to assay gene expression in a snapshot in time (snapshot reporter mouse). The *lacZ* reporter gene is targeted to the endogenous *Vwf* locus. LacZ expression in the resulting vWF^+/LacZ^ mice reflects the current transcriptional state of the endogenous *Vwf* promoter. Bottom, LacZ expression in individual endothelial cells (ON in filled circles; OFF in unfilled circles) over time (black arrow: no change in promoter activity; blue arrow: OFF→ON promoter transition; grey arrow: ON→OFF promoter transition). (**d**) Top, Schematic representation of the gene targeting strategy for generating a fate-mapping mouse model that allows for assessment of cumulative expression over time (cumulative reporter mouse). Cre recombinase is targeted to the endogenous *Vwf* locus. The resulting vWF^+/Cre^ mice are then bred to the ROSA26R reporter line, in which the *lacZ* gene has been targeted to the ubiquitously expressing ROSA26 locus that contains the functional equivalent of a stop codon, flanked by *loxP* sites. In double transgenic offspring (vWF–Cre–ROSA26R mice), cells that express from the *Vwf* promoter result in Cre-mediated excision of the stop codon and permanent expression of LacZ in that cell and all of its progeny. Thus, LacZ expression reflects the cumulative (present and past) transcriptional state of the endogenous promoter. Bottom, LacZ expression in individual endothelial cells (ON/OFF, filled/unfilled circles) over time (black arrow: no change in promoter activity; blue arrow: OFF→ON promoter transition, leading to LacZ expression; grey arrow: ON→OFF promoter transition, masked by locked-in LacZ expression.

The finding of mosaic expression raised the question of how the pattern plays out over time. If we were to ‘roll the film', would the mosaic remain static, indicative of developmentally locked-in hotspots of VWF activity? Alternatively, does expression toggle ON and OFF in time asynchronously between individual endothelial cells? An ideal approach for confirming such dynamicism would be to directly observe VWF protein (or a fluorescent reporter) in real time in the living organism. However, such a strategy is not feasible given current limitations in the resolution of intravital microscopy, combined with the need for potentially long windows of observation. Thus, we used an indirect approach to capture the temporal dynamics of VWF expression. Specifically, we generated mice in which Cre recombinase was targeted to the endogenous *Vwf* locus. These animals (vWF^Cre/+^) were crossed with the ROSA26R reporter mouse line, which contains a LoxP-flanked STOP cassette upstream of the *lacZ* gene in the constitutively active Rosa26 locus. In double transgenic offspring (vWF–Cre–ROSA26R; Supplementary Fig. 1b), cells that express vWF-Cre (even transiently) excise the stop codon in the reporter allele, leading to permanent and heritable expression of LacZ. Thus, reporter gene activity reflects cumulative expression over the lifetime of the animal, and a comparison of LacZ expression between vWF^LacZ/+^ and vWF–Cre–ROSA26R mice provides critical information about the temporal dynamics of VWF expression (Fig. 1c,d).

**Figure 2:**
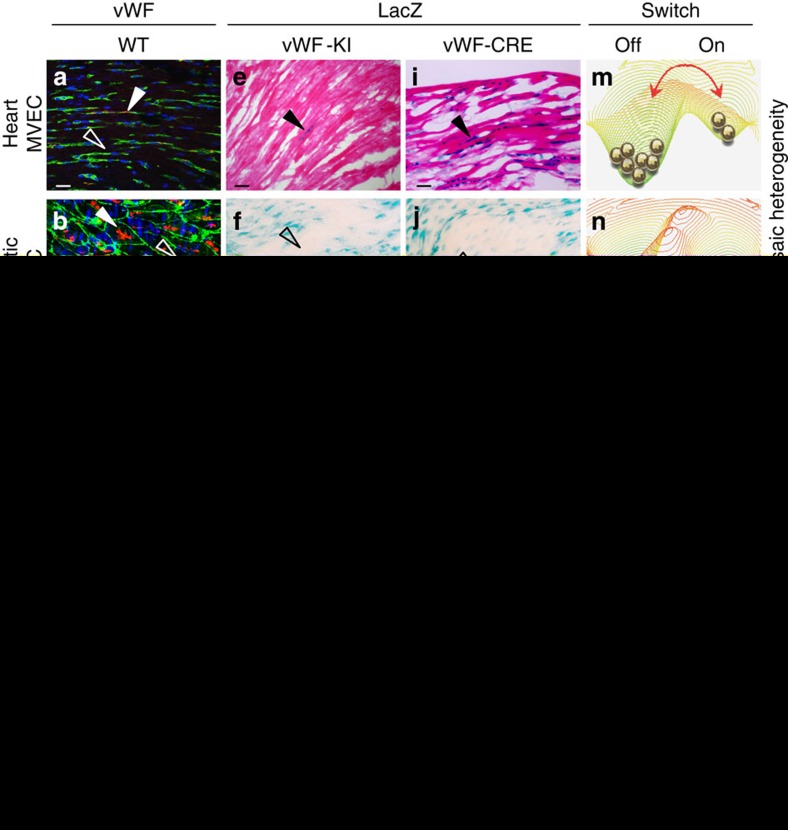
Organ-specific mosaic heterogeneity of VWF *in vivo*. Double immunofluorescence for VWF (red) and CD31 (green) of tissue sections and en face aortae from adult male wild-type (WT) mice (**a**–**d**), and LacZ staining of tissue sections and en face aortae from adult male vWF^LacZ/+^ (vWF knockin or vWF-KI) mice (**e**–**h**) and vWF–Cre–ROSA26R (vWF–CRE) mice (**i**–**l**). (**a**,**e**,**i**) Heart sections with capillaries cut longitudinally. (**b**,**f**,**j**) The inner lining of an aorta that has been opened and laid flat under a coverslip. (**c**,**g**,**k**) Heart sections with capillaries and veins cut in cross section. (**d**,**h**,**l**) Liver sections with hepatocytes surrounded by sinusoids (arrows). (**a**–**l**) VWF- or LacZ-expressing cells are indicated by solid arrows; non-expressing cells are indicated empty arrows; and LacZ-expressing hepatocytes are indicated by an *. Right column (**m**–**p**) shows our proposed model of a bistable switch regulating VWF expression, dynamically toggling between ON and OFF in heart capillaries (**m**), frozen into one of two states in the aorta (**n**) or locked into a single ON or OFF state in heart veins and liver sinusoidal endothelial cells, respectively (**o**,**p**). (**q**–**s**) Whole-mount LacZ staining of heart, muscle (diaphragm) and brain from adult male vWF^LacZ/+^ (vWF-KI) mice and vWF–Cre–ROSA26R (vWF–CRE) mice. Scale bars, (**a**–**i**) 100 μm; (**q**–**s**) 3 mm. *n*=3 with three replicates.

Remarkably, adult vWF–Cre–ROSA26R mice demonstrated uniform LacZ staining in the capillaries of heart, skeletal muscle, lung and brain (Fig. 2i,q–s; Supplementary Fig. 2), suggesting that virtually every capillary endothelial cell had expressed LacZ (hence VWF) at some point during the lifetime of the animal. Since only a minority of these endothelial cells are positive at a single point in time, the data suggest that VWF flickers ON and OFF over time. Interestingly, LacZ expression in the aorta of adult vWF–Cre–ROSA26R mice remained patchy (Fig. 2j; Supplementary Fig. 3), suggesting that the mosaic pattern in this blood vessel is static. Finally, LacZ staining was undetectable in kidney glomerular and liver sinusoidal endothelial cells (Fig. 2l shows liver), indicating that VWF is permanently OFF at these two sites. Together, the data support the notion that VWF toggles between ON/OFF states in some, but not all vascular beds. This behaviour is consistent with the existence of a bistable switch whose state-switching dynamics vary across the vascular tree, as illustrated by the quasi-energy landscapes in Fig. 2m–p.

### VWF mosaicism is dynamically regulated in adult capillaries

**Figure 3:**
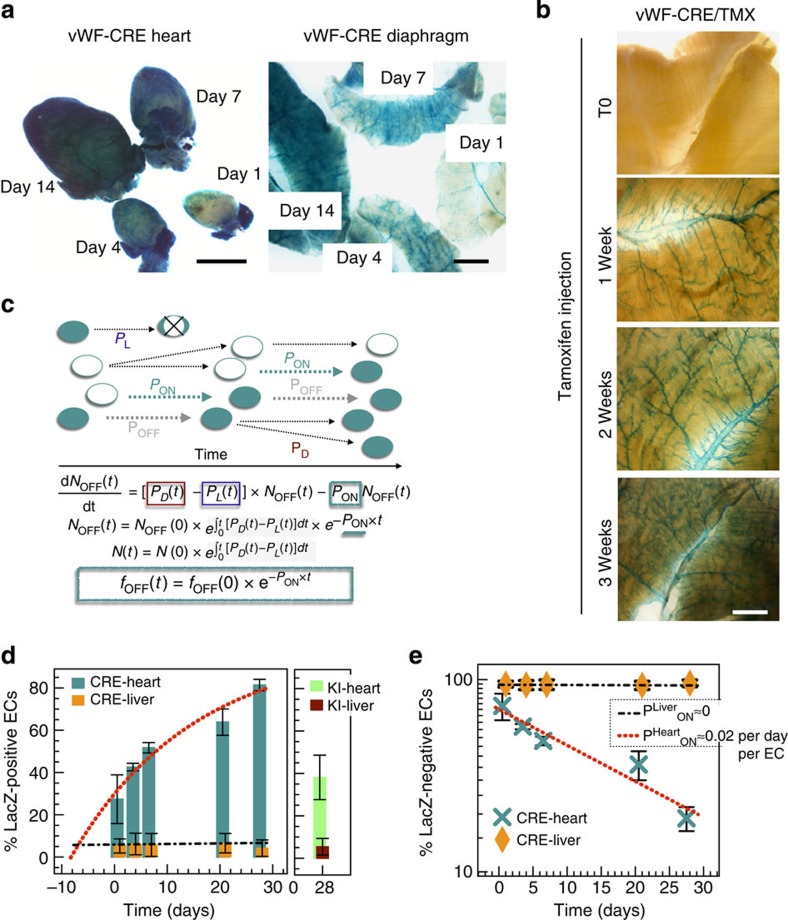
VWF ON/OFF transitions occur in adult endothelium. (**a**) Whole-mount LacZ staining of vWF–Cre–ROSA26R (vWF-CRE) hearts and diaphragms collected at indicated time points following birth (stained together and imaged side by side). Scale bar, 5 mm. (**b**) Time course of whole-mount diaphragm LacZ staining from tamoxifen-treated vWF–CreERT2–ROSA26R (vWF–CRE/TMX) mice. Time 0 (T0) indicates no exposure to tamoxifen. The results reveal time-dependent accumulation of LacZ in the capillaries, which are arranged in linear arrays parallel to the plain of the tissue. Scale bar, 2.5 mm. (**c**) Population dynamics of LacZ-expressing cell accumulation in vWF–Cre–ROSA26R endothelial cells (ECs), modelled by assuming random OFF→ON transitions with constant, division-independent rate *P*_ON_, time-dependent cell division with rate *P*_D_(*t*) and cell loss with rate *P*_L_(*t*). As indicated, the fraction of LacZ-negative cells, *f*_OFF_, decreases exponentially with time (see Supplementary Note 1 for further detail). Teal circle: LacZ-positive cell; white circle: LacZ-negative cell; circle with X: dead cell; black arrow: no promoter transition; blue arrow: OFF→ON transition of the endogenous promoter; grey arrow: ON→OFF transition of the endogenous promoter (masked by locked-in LacZ expression). (**d**) Left, Time-dependent LacZ accumulation in vWF–Cre–ROSA26R (vWF–CRE) mouse heart and liver capillaries (LacZ/CD31 double stained sections); Right, LacZ-positive EC percentage in heart and liver capillaries of 4-week-old vWF^+/LacZ^ mice (knockin, KI). (**e**) Log-linear plot of the LacZ-negative EC fraction, fitted by an exponential with rate *P*_ON_=0.02 per day per EC for heart, and *P*_ON_=0 per day per EC for liver. *n*=3 with three replicates.

The comparative analysis of vWF^LacZ/+^ and vWF–Cre–ROSA26R mice does not prove that VWF mosaicism is dynamic in the postnatal period. It is formally possible that the uniform LacZ staining in capillaries of adult vWF–Cre–ROSA26R mice reflects a developmental window in which a common ancestor of all endothelial cells expresses VWF. To rule this out, we monitored LacZ expression in vWF–Cre–ROSA26R mice from birth to adulthood. Analysis of tissues at postnatal days (P) 1, 4 and 7 and in adult mice revealed a time-dependent LacZ accumulation in the capillary beds of heart, skeletal muscle, lung and brain (Fig. 3a shows heart and diaphragm). In contrast, a similar binary mouse model in which the pan-endothelial Tie2 promoter drives Cre expression demonstrated uniform LacZ staining of blood vessels in both newborn and adult animals (Supplementary Fig. 4a–f).

To explicitly demonstrate that transitions between VWF-positive and -negative cells take place in adult tissues, we generated a mouse model in which we could control the timing of Cre recombinase expression from the *Vwf* locus. Specifically, we targeted tamoxifen-inducible Cre recombinase to the endogenous *Vwf* locus (vWF^CreERT2/+^) and crossed these mice with the ROSA26R reporter line (Supplementary Fig. 1c). Double-transgenic mice (vWF-CreERT2-ROSA26R) were treated with tamoxifen for increasing lengths of time (up to 4 weeks), and their organs were harvested for LacZ staining. In the absence of tamoxifen, there was no detectable reporter gene activity. In contrast, mice treated with tamoxifen demonstrated time-dependent LacZ accumulation in veins and capillaries of the heart, skeletal muscle, lung and brain (Fig. 3b shows diaphragm). Again, the aorta demonstrated a fixed mosaic, while there was no detectable expression in the kidney glomeruli or hepatic sinusoids (Supplementary Fig. 4g–i). These data indicate that uniform LacZ staining in adult muscle capillaries reflects the accumulation of transiently expressing (VWF ON/OFF) cells in the postnatal period, and that vascular bed-specific dynamic mosaicism is a property of healthy adult endothelium.

### Dynamic VWF mosaicism is driven by biological noise *in vivo*

A simple explanation for spontaneous toggling between VWF ON and OFF is biological noise. According to this hypothesis, the barrier of the binary switch is sufficiently low to allow for stochastic transitions in the capillaries of heart, skeletal muscle, lung and brain (Fig. 2m), yet high enough to preclude such transitions in the aorta (which displays a static ON/OFF mosaic; Fig. 2n)^4^. If dynamic VWF expression is indeed driven by biological noise with constant average strength, then the number of LacZ-negative cells in vWF–Cre–ROSA26R heart capillaries should decrease exponentially with time: *f*_OFF_(*t*)∼exp(−*P*_ON_ · *t*) (Fig. 3c; *P*_ON_=probability of OFF→ON transitions per unit time per endothelial cell; Supplementary Note 1). To test this, we quantified time-dependent accumulation of LacZ in vWF–Cre–ROSA26R mouse heart and liver capillaries co-immunostained for LacZ and pan-endothelial CD31, from birth to adulthood (Fig. 3d, Supplementary Fig. 4j–u). In line with our predictions, the time-dependent percentage of LacZ-negative endothelial cells in heart capillaries fell along a log-linear fit (turquoise bars/crosses, red curve/line in Fig. 3e). The slope of this fit is a direct measurement of the VWF OFF→ON transition rate, *P*_ON_=0.02±0.003 per day per endothelial cell. As LacZ expression in vWF–Cre–ROSA26R mice cannot turn OFF (even though the *VWF* promoter itself keeps toggling), the VWF ON→OFF rate *P*_OFF_ cannot be measured directly. In adult vWF^LacZ/+^ mice, however, the fraction of LacZ-positive cells, *f*_ON_, represents a dynamic equilibrium between ON and OFF transitions of the same *VWF* promoter: *f*_ON_ · *P*_OFF_=*f*_OFF_ · *P*_ON_. Thus, measuring *f*_ON_=38% in vWF^LacZ/+^ mice allowed us to calculate *P*_OFF_=*P*_ON_·(1−*f*_ON_)/*f*_ON_=0.03 per day per endothelial cell. LacZ accumulation in the diaphragm and skeletal muscle of the chest wall was qualitatively similar to that in the heart (Fig. 3a shows diaphragm). In contrast, an unchanging 94.5% of liver capillary endothelial cells were locked into a VWF-negative state (orange/dark-red bars; orange diamonds in Fig. 3d,e), with *P*_ON_=*P*_OFF_≈0. Together, these findings point to a random noise-driven process, resulting in dynamic mosaic VWF expression.

In addition to ever-present fluctuations in the rates of gene transcription, protein translation and regulatory network activity^14^,^15^,^16^,^17^,^18^,^19^,^20^, intracellular noise can also arise from asymmetric partitioning of a cell's contents during division^39^. If cell division rate plays an important role in driving VWF ON/OFF transitions, *P*_ON_ should be higher in the early postnatal period when the endothelium is rapidly proliferating. However, the log-linear fit on Fig. 3e points to constant *P*_ON_ throughout the time window of observation. Consistent with these observations, LacZ saturation in the adult vWF-CreERT2-ROSA26R mouse occurred on a similar time scale to that observed in young vWF–Cre–ROSA26R mice (Fig. 3b). These findings argue against a significant contribution of cell division to noise-induced VWF ON/OFF transitions.

### Dynamic VWF mosaicism is driven by biological noise *in vitro*

**Figure 4:**
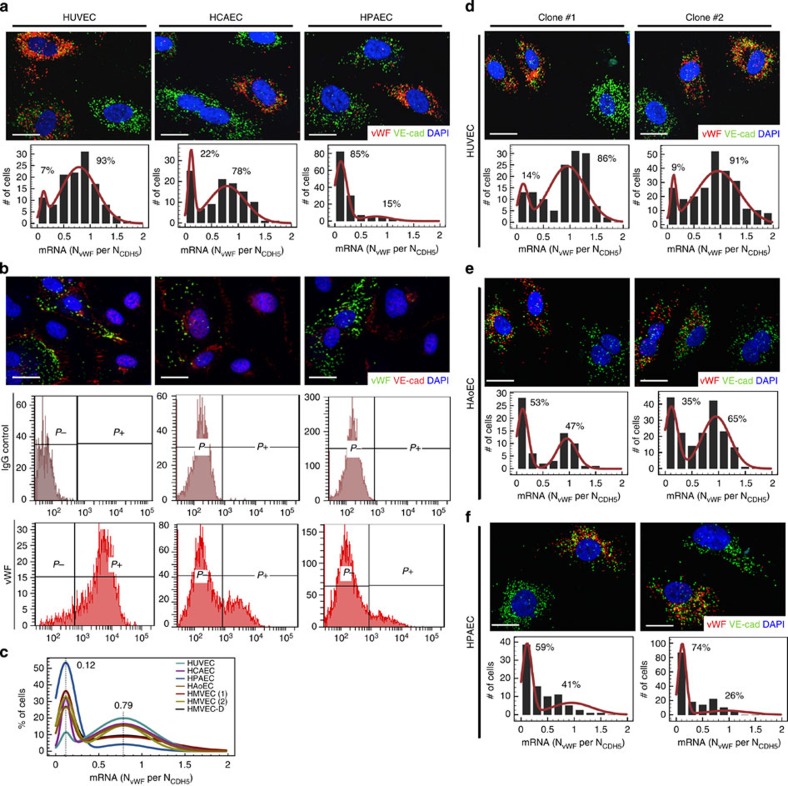
VWF is expressed as a dynamic mosaic in cultured endothelial cells. (**a**) *VWF* and VE-cadherin mRNA distribution in human umbilical vein endothelial cells (HUVEC), human coronary artery endothelial cells (HCAEC) and human pulmonary artery endothelial cells (HPAEC). *Top*, Fluorescence *in situ* hybridization (FISH) for VWF/VE-cadherin (red/green; see Supplementary Fig. 5 for larger fields). Cell nuclei were stained with DAPI (blue). Bottom, *VWF* mRNA distributions in single cells, normalized to VE-cadherin. (**b**) Protein distributions in HUVEC, HCAEC and HPAEC. Top, Immunostaining for VWF/VE-cadherin (green/red). Cell nuclei were stained with DAPI (blue). Middle/bottom, Flow cytometry analysis of IgG control and VWF protein expression (*P−*/*P+*: negative/positive). (**c**) Overlay of fitted *VWF* mRNA distributions from all assayed human endothelial cell (EC) populations: HUVEC, HCAEC, HPAEC, human aortic ECs (HAoEC), human cardiac microvascular ECs (HMVEC) and human dermal microvascular ECs (HMVEC-D; see Supplementary Note 8 for Gaussian mixture fitting methods; Supplementary Table 2 for fit parameters). (**d**–**f**) Clonal populations derived from a single HUVEC (**d**), HAoEC (**e**) or HPAEC (**f**). *Top*, FISH for VWF/VE-cadherin (red/green). Cell nuclei were stained with DAPI (blue). *Bottom: VWF* mRNA distribution normalized to VE-cadherin (see Supplementary Table 3 for fit parameters). (**a**–**f**) Scale bar, 20 μm; *n*=6 with three replicates.

Stochastic transitions between VWF ON and OFF states may arise from random perturbations in the cell's extracellular environment or from the stochastic variation in intracellular processes. To distinguish between these two possibilities, we turned to cell culture where we could more precisely control the extracellular environment. We employed fluorescence *in situ* hybridization (FISH) and immunofluorescence assays to evaluate VWF expression in monolayers of endothelial cells. Similar to our *in vivo* observations, and in contrast to the prevailing dogma that VWF is a universal marker for cultured endothelial cells, we found that VWF expression follows a mosaic pattern in multiple subtypes of primary human endothelial cells (Fig. 4a,b; Supplementary Figs 5 and 6a,b). Flow cytometric analysis of VWF protein and single-cell assays for *VWF* mRNA expression revealed a bimodal distribution in protein and mRNA, respectively. Individual *VWF* mRNA distributions normalized to VE-cadherin are well described by linear combinations of two binomial distributions with cell-type-specific mixing weights (Fig. 4c; fitting detailed in Supplementary Note 2). These findings suggest that VWF expression in cultured endothelial cells is mediated by a bistable ON/OFF switch, resulting in two distinct cell states. To prove that the VWF switch is dynamically regulated, we used limiting dilution to clone single endothelial cells and then expanded each clone to ∼10^6^ cells. All clonally expanded populations demonstrated a mix of VWF low- and high-expressing endothelial cells, with *VWF* mRNA and protein distributions similar to those of the parent population (Fig. 4d–f). It is noteworthy that under *in vitro* conditions, even human aortic endothelial cells demonstrated dynamic mosaicism, in contrast to the robust static mosaic pattern observed in mouse aortas *in vivo*. Together, these findings suggest that biological noise results in stochastic transitions between the ON and OFF states of a bistable VWF switch (alternative mechanisms inconsistent with our data are discussed in Supplementary Note 3).

### DNA methylation correlates inversely with VWF expression

**Figure 5:**
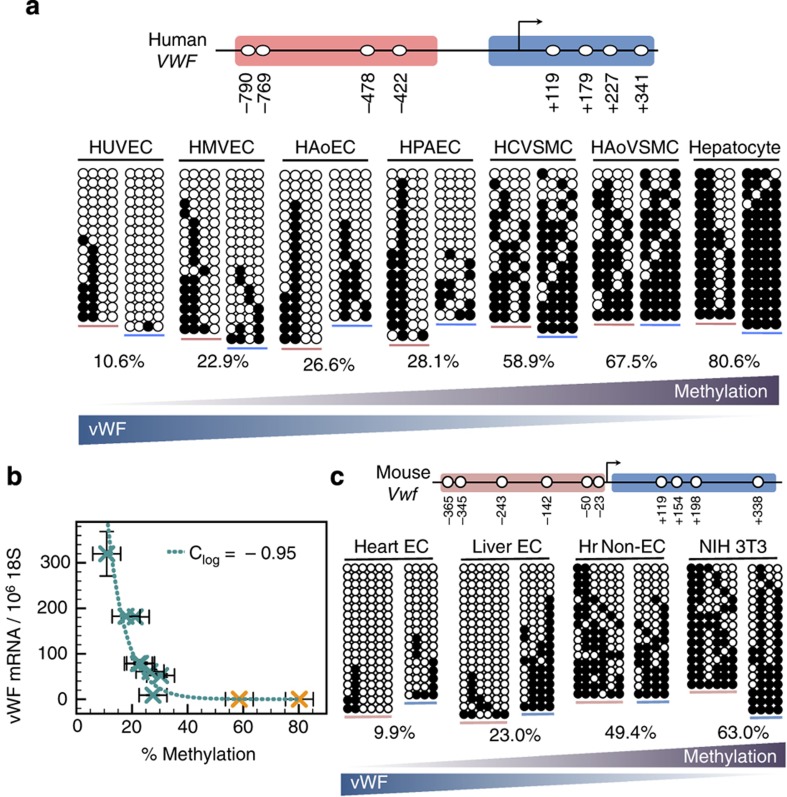
DNA methylation correlates inversely with VWF expression. (**a**) Top, Schematic showing the eight CpG sites in the proximal human *VWF* promoter. Bottom, Bisulfite sequencing of the human *VWF* promoter in four endothelial and three non-endothelial cell populations. HUVEC, human umbilical veins ECs; HMVEC, human cardiac microvascular ECs; HAoEC, human aortic ECs; HPAEC, human pulmonary artery ECs; HCVSMC, human coronary artery vascular smooth muscle cells; HAoVSMC, human aortic vascular smooth muscle cells. Each continuous row of circles represents the upstream/core region of a single *VWF* allele; ●/○: methylated/unmethylated CpG. The upstream/core promoter regions are marked by pink/blue underlines, respectively. The percentages (here as well as in **b**–**c**) refer to methylated/total CpG for all eight CpG sites. (**b**) *VWF* promoter DNA methylation versus *VWF* mRNA levels (shown relative to 10^6^ 18S copies) in endothelial (teal X) and non-endothelial (orange X) cell populations (for methylation error estimates see Supplementary Note 3 and Fig. 7a). Teal dashed line shows a log-normal fit, indicating that *VWF* mRNA expression decreases exponentially with DNA methylation. Vertical error bars indicate standard deviation of mRNA expression; horizontal error bars indicate estimated DNA methylation sampling error (see Supplementary Note 4 and Fig. 7e). (**c**) Top, Schematic showing the CpG sites in the proximal mouse *Vwf* promoter. Bottom, Bisulfite sequencing of the mouse *Vwf* promoter in two endothelial and two non-endothelial cell populations. Hr non-EC represents the non-endothelial cell (EC) fraction from mouse heart (Hr) preparations. All experiments performed with three replicates.

Since the *in vivo* rate of switching is on the order of weeks, we posited that the tight OFF state is maintained by epigenetic modification of the promoter^40^,^41^,^42^. DNA methylation of promoter-region CpG dinucleotides is a potent epigenetic mechanism for silencing transcription^40^. The human *VWF* promoter contains eight CpGs between nucleotide positions −900 and +400, relative to the transcriptional start site (Fig. 5a). We hypothesized that the methylation of one or more of these sites is an important determinant of the ON/OFF state of the gene. To test this, we carried out sodium bisulfite genomic sequencing in several endothelial and non-endothelial cell types. This technique determines the methylation status of the eight CpG sites in the *VWF* promoter at the level of a single allele. For each cell type, we carried out bisulfite sequencing on at least 15 random *VWF* alleles. As shown in Fig. 5a, the *VWF* promoter was lightly methylated in multiple types of human endothelial cells (11–28% of the eight CpG sites), but heavily methylated in VWF-non-expressing cells, including vascular smooth muscle cells and hepatocytes (59–81% of the eight CpG sites). Overall, VWF expression correlated inversely with the average methylation of the eight CpGs (Fig. 5b; see Supplementary Fig. 7a and Note 4 for methylation sampling error estimates). Similar findings were observed with the mouse *Vwf* promoter (Fig. 5c). In agreement with the expected link between DNA methylation and chromatin configuration^43^,^44^, we found a positive correlation between VWF expression levels and a transcriptionally permissive chromatin structure (Supplementary Fig. 7b–d). These findings suggest that both promoter DNA methylation and chromatin mechanisms contribute to differential expression of VWF across various cell types.

**Figure 6:**
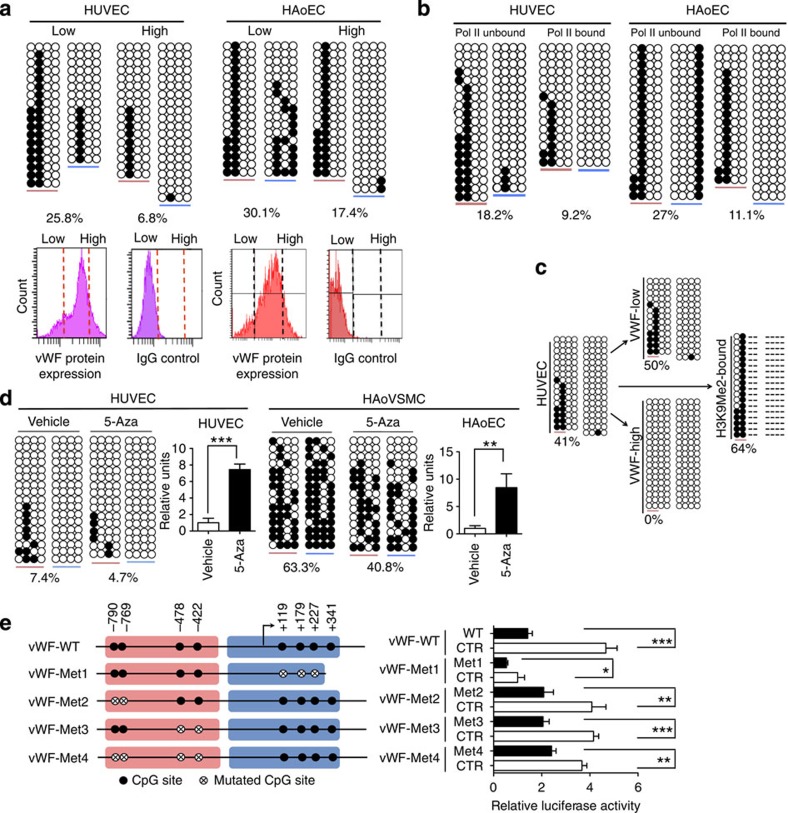
DNA methylation is necessary and sufficient for silencing VWF expression. (**a**) Top, Bisulfite sequencing of the human *VWF* promoter in endothelial cells (ECs) that were FACS-sorted into subpopulations that expressed low levels of VWF (low) and cells that expressed high levels of VWF (high). Bottom, FACS profiles of VWF expression (left) and IgG control (right). The vertical red and black lines indicate the gating thresholds used for sorting. (**b**) Pol II ChIP assay followed by bisulfite sequencing of Pol II-bound and -unbound chromatin in human umbilical vein ECs (HUVEC) and human aortic ECs (HAoEC). (**c**) Repressive histone H3 lysine 9 dimethylation (H3K9Me2) ChIP assay followed by bisulfite sequencing of H3K9Me2-bound chromatin in HUVEC. Here, percentages denote the fraction of methylated CpGs at the first two CpG sites; dashes indicate unsequenced CpG sites due to lack of H3K9Me2 pull-down. (**d**) *VWF* mRNA expression and *VWF* promoter methylation following 5-azacytidine (5-Aza) treatment in HUVEC and HAoVSMC. *VWF* mRNA expression levels are shown relative to untreated vehicle controls. (**e**) Luciferase assay for methylated versus unmethylated *VWF* promoter/reporter constructs. *Left*: generation of 5′ *VWF* promoter/reporter constructs with mutations (CG→TG) at indicated CpG sites (●/⊗: non-mutated/mutated CpG sites); *VWF* promoter/reporter constructs were *in vitro* methylated using SssI methylase. Right: Luciferase activity in HUVEC transiently transfected with mock-methylated (white) or methylated (black) VWF constructs or pGL3 basic (units normalized to pGL3). HAoVSMC, human aortic vascular smooth muscle cells; Error bars indicate s.d.; *n*=3, with three replicates. Two-sided *T*-test was performed in all significance tests. *P<0.05; **P<0.01; ***P<0.001.

To determine whether differences in DNA methylation are also involved in mediating mosaic expression of VWF within a population of isogenic cells, we employed flow-activated cell sorting (FACS) to isolate single-donor human umbilical vein endothelial cells (HUVEC) with the highest (top 10%) and lowest (bottom 10%) levels of VWF and compared their DNA methylation using bisulfite genomic sequencing. As shown in Fig. 6a (left), the *VWF* promoter was significantly hypomethylated in the VWF-high subpopulation, compared with the VWF low fraction. Similar results were observed with human aortic endothelial cells (Fig. 6a, right). To provide a more direct link between DNA methylation and *VWF* transcription, we used antibodies against RNA polymerase II to immunoprecipitate transcriptionally active chromatin fragments in endothelial cells, and then carried out bisulfite sequencing on the Pol II-bound and -unbound chromatin fractions. These experiments showed that the Pol II-bound *VWF* alleles were consistently and significantly less methylated than the transcriptionally inactive Pol II-unbound *VWF* alleles (Fig. 6b). Moreover, DNA hypermethylated *VWF* alleles were associated with repressive histone H3 lysine 9 dimethylation (Fig. 6c).

To determine a causal link between VWF expression and DNA methylation, we carried out two additional assays. First, we measured VWF expression in various cells in the absence or presence of the DNA methyltransferase inhibitor 5-azacytidine (5-Aza). As shown in Fig. 6d, 5-Aza partially demethylated the *VWF* promoter, resulting in a seven- to eightfold increase in *VWF* mRNA expression in endothelial cells and *de novo* expression in otherwise VWF-negative vascular smooth muscle cells. Second, we used M.SssI to methylate CpG sites in the human *VWF* promoter (coupled to the *lacZ* reporter gene) and transfected the resulting episomal plasmid into human endothelial cells. Compared with the mock-methylated control, the *in vitro* M.SssI-methylated plasmid demonstrated significantly less promoter activity (Fig. 6e). Similar results were observed when only the proximal (Met 4) or the distal (Met 1) CpG sites were methylated. Taken together, these findings suggest that DNA hypermethylation of the *VWF* promoter is a repressive mark that is both necessary and sufficient for the VWF OFF state. Conversely, hypomethylation of the *VWF* promoter is required for the VWF ON state.

### VWF DNA methylation spontaneously toggles ON and OFF

**Figure 7:**
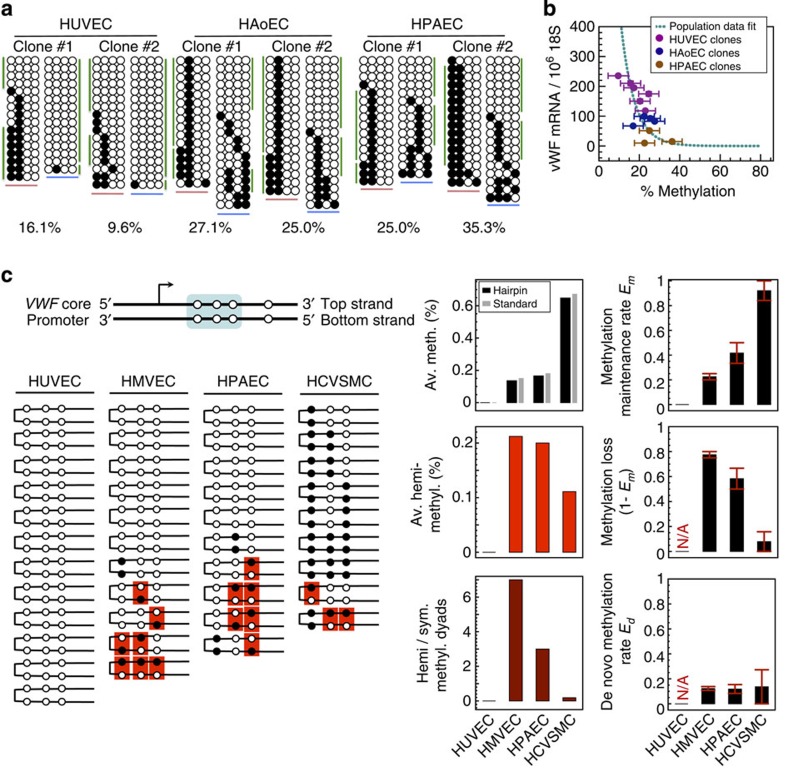
DNA methylation of the *VWF* promoter undergoes random transitions. (**a**) Bisulfite sequencing of the human *VWF* promoter in clonally expanded endothelial cells (ECs). Each continuous row of circles represents the upstream/core region of a single *VWF* allele; ●/○: methylated/unmethylated CpG. The upstream/core promoter regions are marked by pink/blue underlines, respectively. The vertical green lines indicate the two dominant DNA methylation patterns, which represent the most likely methylation patterns of the two *VWF* alleles in the original single cell for an estimate of observing similar heterogeneity due to experimental error, see Supplementary Note 5). Percentages refer to methylated/total CpG across all eight CpG sites. *n*=5, with three replicates. (**b**) *VWF* promoter DNA methylation versus *VWF* mRNA expression in clonally expanded ECs. Multiple independently derived clonal populations were bisulfite sequenced for each EC type (for methylation sampling error estimates see Supplementary Note 4). Teal dashed line shows the log-normal dependency of *VWF* mRNA expression on average DNA methylation, derived from the parent EC and non-EC populations shown in Fig. 5b. Purple: human umbilical vein ECs (HUVEC); blue: human aortic ECs (HAoEC); brown: human pulmonary artery ECs (HPAEC). (**c**) Top, Schematic showing the three out of four CpG sites in the proximal human *VWF* promoter that were assayed by hairpin bisulfite sequencing (blue background). Middle left, Hairpin bisulfite sequencing of the three CpG sites in multiple EC types as well as human coronary artery smooth muscle cells (HCVSMC). Middle right, average methylation (black, hairpin bisulfite; *grey*, standard bisulfite from Fig. 5a), average hemi-methylation (red, hemimethylated CpG dyads/all dyads) and the fraction of hemimethylated versus fully symmetrically methylated dyads (dark red). Bottom, rates of methylation maintenance (*E*_m_), methylation loss (1−*E*_m_), and *de novo* methylation (*E*_d_) (error bars: minimum-maximum estimate; N/A: not applicable; see Supplementary Note 6). HMVEC, human cardiac microvascular ECs.

The above results raise the interesting possibility that dynamic regulation of DNA methylation is required for the noise-induced transitions between VWF ON and OFF states. To test this hypothesis, we performed bisulfite sequencing on clonal populations derived from single endothelial cells. In these experiments, we observed a mix of more than two methylation patterns in each assayed clone (Fig. 7a), indicating that the original pattern of DNA methylation of the two parental *VWF* alleles was not faithfully maintained (the likelihood of observing these CpG patterns dues to experimental error is estimated in Supplementary Note 5 and Fig. 7e). Moreover, average VWF methylation was inversely correlated with VWF expression across independently derived clones (Fig. 7b). Overlay of these data onto the exponential fit from Fig. 5b, representing the methylation–expression relationship seen in parent endothelial cells and non-endothelial cells, demonstrated a similar relationship to that observed in parent cell lines (Fig. 7b). To directly prove the occurrence of ongoing *VWF* DNA methylation/demethylation, we performed hairpin bisulfite sequencing on three CpG sites in the core *VWF* promoter (Fig. 7c, top). This technique uses a short hairpin to link the top and bottom complementary strands of a double-stranded DNA near a region of interest, allowing for determination of the methylation state of a C nucleotide on both sides of a single CpG/CpG dyad^45^,^46^. Any mismatch between methylation states of the two CpGs in a dyad is indicative of ongoing DNA methylation/demethylation. As shown in Fig. 7c, the methylation status was relatively stable in cells at the highest and lowest ends of VWF expression, namely in HUVEC, which demonstrate high-average VWF expression and low/absent methylation at the core promoter, and in human coronary artery vascular smooth muscle cells (HCVSMC) in which *VWF* is transcriptionally silent and heavily methylated. In contrast, we observed a high incidence of hemimethylated dyads in human cardiac microvascular endothelial cells (HMVEC) and human pulmonary artery endothelial cells (HPAEC), which express medium levels of VWF. Following the stochastic model of DNA methylation proposed in ref. 47, we used these data to estimate the rates of methylation maintenance (*E*_m_), demethylation (1−*E*_m_) and *de novo* methylation (*E*_d_) in different cell types (Supplementary Note 6). Endothelial cells with a high incidence of hemimethylated CpG dyads demonstrated higher rates of DNA methylation loss (failure of maintenance and/or increased demethylation; 0.56 per CpG per division in HPAEC and 0.71 per CpG per division in HMVEC) compared with HCVSMC (0.1 per CpG per division; Fig. 7c). Together, these findings suggest that both VWF expression and CpG methylation flickers ON and OFF in isogenic cells.

**Figure 8:**
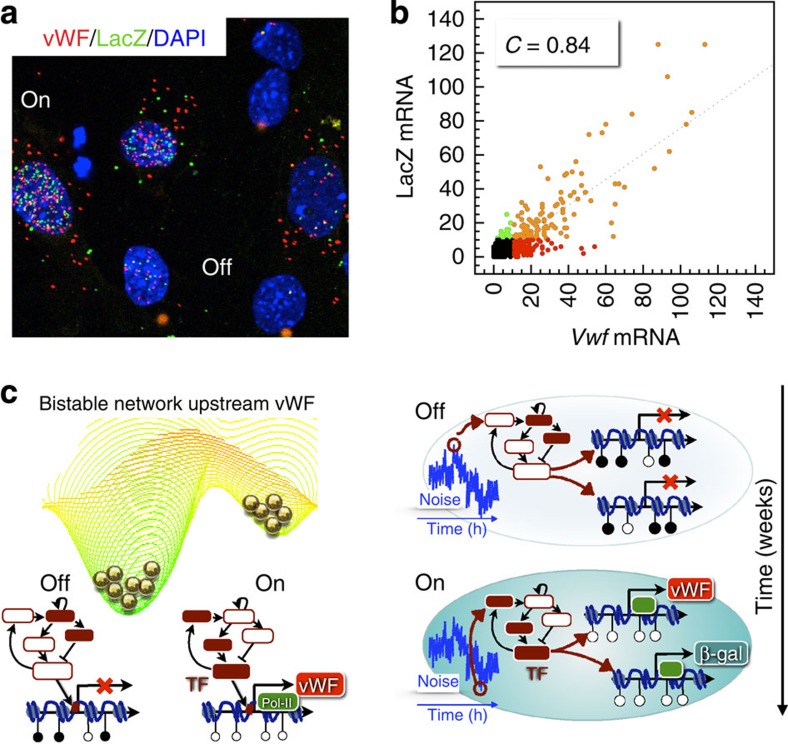
Coupled activity of the two VWF alleles in cultured endothelial cells. (**a**) FISH co-localization of *Vwf* (red) and *lacZ* (green) mRNA in mouse heart microvascular endothelial cells (ECs) harvested from vWF^+/LacZ^ mice. Cell nuclei were stained with DAPI (blue). Scale bar, 20 μm. (**b**) Quantitation of *lacZ* versus *Vwf* mRNA in single cells from **a** shows strong positive correlation with *C*=0.84 (*n*=804 ECs). Black dots: ECs with less than 10 copies of both *Vwf* and *lacZ*; red dots: ECs with more/less than 10 copies of *Vwf*/ *lacZ*; green dots: ECs with less/more than 10 copies of *Vwf*/*lacZ*; orange dots: ECs with more than 10 copies of both *Vwf* and *lacZ*. (**c**) Schematic summary of the mechanism of *Vwf* bistability. Left, Landscape of a bistable regulatory circuit converging on the *Vwf* promoter. Red/white nodes of small network: schematic representation of high/low activity of transcription factors and/or signalling molecules in the upstream circuit; red box: transcription factor/epigenetic modifier binding site on the *Vwf* promoter; ●/○: methylated/unmethylated CpG. Right, OFF/ON VWF cell states. The state of a yet unidentified, bistable but noise-sensitive upstream circuit dictates both DNA methylation status and transcriptional activity of the *Vwf* promoter, toggling both alleles in concert (blue curve: intracellular noise). *n*=5, with three replicates.

We next asked whether DNA methylation constitutes the binary switch itself^48^,^49^ (please see Supplementary Note 7 for an explanation of how DNA methylation may theoretically create such a switch), or whether it simply ‘marches to the tune' of an upstream noise-sensitive bistable switch. We reasoned that if DNA methylation (with associated changes in chromatin configuration) is sufficient for the switch-like behaviour of VWF, then the two *Vwf* alleles should toggle independently. If, on the other hand, the switch is located in an upstream regulatory pathway, the two alleles should be co-regulated. To test this, we isolated cardiac microvascular endothelial cells from vWF^LacZ/+^ mice (in which one *Vwf* allele drives VWF expression, and the other drives LacZ expression) and carried out co-FISH staining for VWF and LacZ (Fig. 8a). As shown in Fig. 8b, there was a strong correlation between *Vwf* and *lacZ* mRNA numbers in individual cells, arguing against independent switching of the two alleles and in accord with previous reports for other loci^50^,^51^. Instead, the coupled activity of the two alleles suggests that the relevant noise source is upstream of the *Vwf* promoter, and that dynamic VWF mosaicism is driven by a bistable regulatory network that converges on the transcriptional and/or DNA methylation machinery at the *Vwf* promoter (Fig. 8c).

### VWF expression is required for cardiac health

An important question is whether stochastic transitioning between VWF ON/OFF states is functionally relevant. To address this, we focused on the adult heart. We reasoned that if mosaic VWF expression in the heart has an adaptive role, then loss of expression from the minority of normally VWF-positive cells in VWF-null mice might lead to a cardiac phenotype. As discussed in Supplementary Note 8 and shown in Supplementary Figs 8 and 9, VWF-null mice demonstrated marked abnormalities in capillary endothelial cells from the right and left ventricles. Moreover, cardiac function was impaired. Together, these findings indicate that VWF expression is necessary for cardiac health. They raise the possibility, but do not prove, that dynamic mosaicism in heart capillaries is responsible for this effect.

We next asked what mechanism might be responsible for the deleterious effect of VWF knockout on cardiac health. Based on the well-recognized role of VWF in mediating platelet adhesion to the subendothelial surface of injured blood vessels^5^,^52^, we anticipated that the cardiac phenotype in VWF-null mice would be attributed to focal microhemorrhaging in the capillary bed. However, we could not detect any evidence of red blood cell extravasation or plasma leak in the heart. Interestingly, recent studies have implicated non-haemostatic roles for VWF^53^,^54^,^55^, including the modulation of angiopoietin-Tie signalling^56^. We present data suggesting that dynamic mosaicism of VWF expression in the capillaries of the heart is associated with randomly shifting hotspots of sustained Ang2 secretion, and that disruption of the mosaic leads to dysregulated Ang2 release and secondary microvascular damage (Supplementary Note 9 and Fig. 10).

### Dynamic mosaicism is not limited to VWF

**Figure 9:**
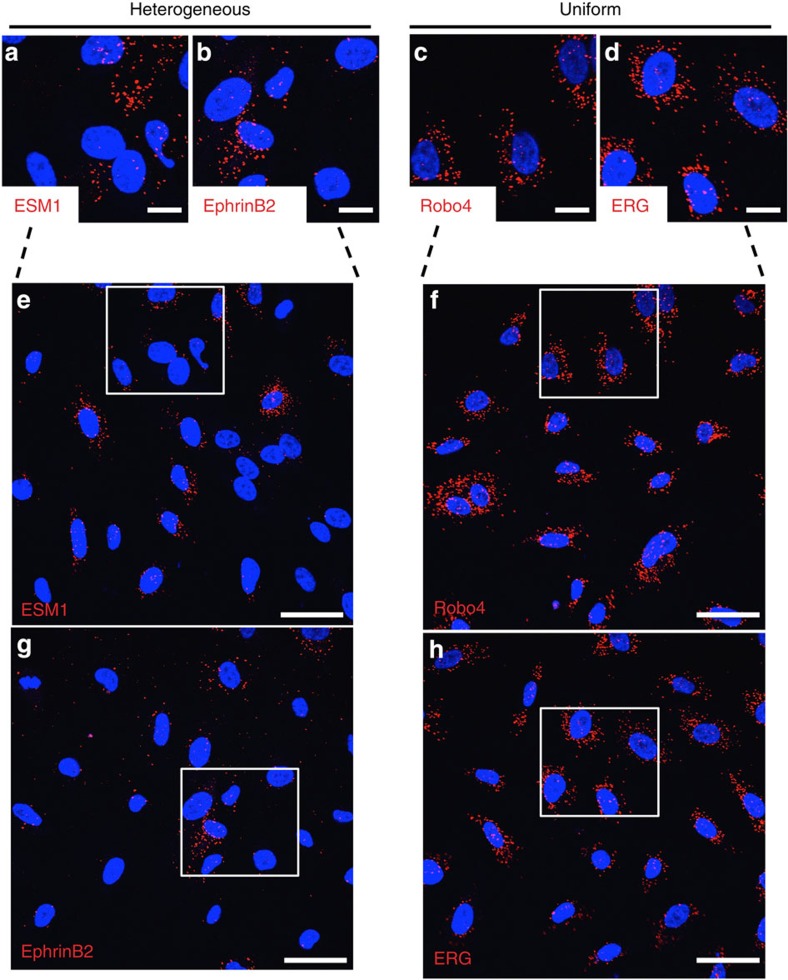
Dynamic mosaic heterogeneity is not restricted to VWF. FISH for endothelial-specific molecule 1 (ESM1), Ephrin B2, Roundabout 4 (Robo4) and ETS-related gene (ERG) in clonal human umbilical vein endothelial cells expanded from a single endothelial cell (EC). ESM1 and Ephrin B2 display mosaic mRNA heterogeneity, while Robo4 and ERG are uniformly expressed in all ECs. Red: ESM1/EFNB2/Robo4/ERG; blue: DAPI. *n*=5, with three replicates. Scale bar, (**a**–**d**) 20 μm; (**e**–**h**) 40 μm.

To determine whether the dynamic mosaic heterogeneity observed with *Vwf* is limited to that gene or represents a more generalized phenomenon in endothelial cells, we carried out FISH analysis of four different endothelial-restricted genes in parent and clonal populations of cultured human endothelial cells: endothelial-specific molecule 1 (*ESM1*, important for sprouting angiogenesis^57^); ephrin-B2 (ref. 58) and *ROBO4* (ref. 59) (involved in vascular patterning); and the endothelial-specific transcription factor *ERG*^60^. These experiments revealed mosaic mRNA expression of *ESM1* and ephrin-B2 in parental and clonal populations of isogenic cells, and uniform expression of *ROBO4* and *ERG* (Fig. 9). These findings suggest that dynamic mosaic heterogeneity is not unique to VWF, and may represent a more widespread adaptive behaviour of the endothelium.

## Discussion

**Figure 10:**
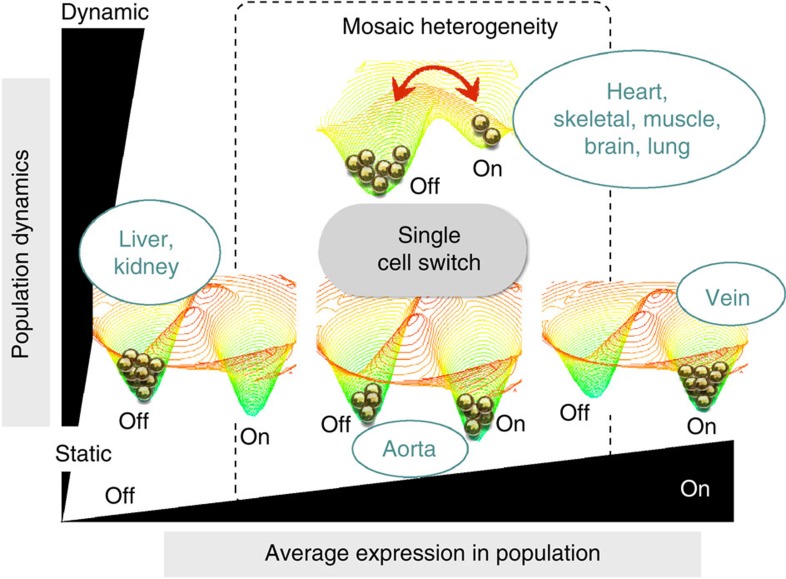
Two dimensions of bistable VWF regulation. Intra- and extracellular environments can fine-tune bistable regulatory switches by setting the percentage of cells in each state (*x* axis), and/or the transition rate between states (and thus the significance of biological noise; *y* axis). In principle, any bistable regulatory switch can be placed into an environment that allows for mosaic heterogeneity. Thus, organ-specific microenvironments can tune the importance of noise relative to the barrier height of a biological switch, above and beyond setting the percentage of cells in each state. In case of VWF, certain tissue and/or intracellular environments strongly regulate the percentage of VWF-positive cells by locking the switch ON (vein) or OFF (kidney). Other environments, such as those in heart muscle versus the aorta, alter the barrier of the switch, giving rise to a dynamic or static mix of VWF ON and OFF cell states.

Mechanisms of endothelial heterogeneity are often attributed to differences in the extracellular environment (‘nurture') or to memory of past differences encoded by epigenetic modifications (‘nature')^3^,^10^. However, a different mechanistic framework is required to explain dynamic mosaicism, in which endothelial phenotypes spontaneously flip in adjacent cells exposed to near-identical microenvironments. To this end, we turned to dynamical systems theory to reframe endothelial cell heterogeneity in terms of multistability^4^, the ability of single cells to maintain distinct phenotypes under identical extracellular conditions (the ‘nature' side of the equation). Multistability is generated through positive regulatory feedback and/or epigenetic modifications that generate barriers between distinct states. These states may be represented as valleys on a landscape^4^,^11^,^21^,^22^, with the internal state of an endothelial cell represented as a marble on its surface (Fig. 1). Endothelial cells constantly navigate this landscape in response to extracellular signals, as well as intracellular noise. When exposed to appropriate signals, cells may cross barriers and enter other valleys (the ‘nurture' side of the ledger). Biological noise results in pervasive cell state fluctuations within a valley. When barriers are sufficiently low, as in the case of VWF in capillary endothelial cells of heart, skeletal muscle, lung and brain, noise leads to stochastic transitions between two distinct phenotypes. Consequently, the mosaic pattern ‘blinks' ON/OFF during the lifetime of a cell (Fig. 10). Correlated expression of the two VWF alleles (Fig. 7a,b) suggests that stochastic ON/OFF transitions of the promoter are mediated not by stochastic interactions between RNA polymerases and DNA (that is, intrinsic noise)^20^, but rather by upstream (i.e., extrinsic) noise that converges on both alleles^14^. Since extrinsic noise typically affects the expression of several genes, it is likely that the vWF ON/OFF states are associated with distinct transcriptional profiles. In future studies, it will be interesting to identify the origin of the extrinsic noise responsible for toggling vWF expression, and to identify those genes that are co-regulated with vWF.

Biological noise provides a source of cell-type diversification in clonal populations, and can confer a fitness advantage to cell collectives or an organism. In bet hedging, unicellular organisms stochastically commit some fraction of their population to a phenotype that anticipates the arrival of a new environment, for example, metabolic stress, starvation or antibiotics^24^,^25^,^27^. This commitment is often dynamic: cells randomly transit between alternate states, thus avoiding the slow response-time of a sensory apparatus^27^,^61^,^62^. Alternatively, noise can generate phenotypic specialization within a cell population^28^,^29^ (task allocation), thereby aiding resource utilization. In multicellular organisms, stochastic gene expression can trigger cell-fate decisions such as retinal and olfactory receptor patterning^31^,^32^ or hematopoietic cell differentiation^30^. Here, once a cell makes a stochastic choice towards a new fate, that cell and its progeny remain locked in, resisting further noise-driven change. The dynamic switch-like behaviour of VWF expression in capillaries of the heart, skeletal muscle, lung and brain is more reminiscent of bet hedging in unicellular organisms than it is of irreversible noise-induced cell-fate decisions observed in higher eukaryotes (Fig. 9). By contrast, the static VWF mosaic in the aorta suggests that while the initial patterning during development may have been initiated by stochastic decision-making, the new state (ON or OFF) has become locked in.

Our findings raise the interesting question of whether noisy expression of VWF is adaptive and if so, how. It is possible that the flickering nature of VWF expression is a byproduct of molecular noise with no effect on fitness. Since noise suppression is an active and costly process^62^,^63^,^64^, such a neutral flickering in VWF expression would not be selected against. Alternatively, the stochastic transitions in VWF expression may be a byproduct of co-regulation of another gene that provides a selective advantage. A final explanation, which we favour, is that VWF switching itself is advantageous. In support of this conclusion, we found that VWF-null mice have a markedly abnormal endothelial phenotype in capillaries of the heart. VWF is a haemostatic protein that mediates platelet adhesion to the blood vessel wall. Too little VWF predisposes to a bleeding phenotype, whereas excessive—or perhaps uniform—expression of VWF may promote clotting. Stochastic expression of VWF might serve a middle ground, by allowing a constantly shifting subpopulation of capillary endothelial cells to anticipate and respond to vascular injury; analogous to bet hedging in a colony of genetically identical unicellular organisms.

Recent studies have shown that VWF has certain non-haemostatic functions. For example, in the mouse brain, VWF was shown to attenuate the blood–brain barrier under stress conditions by locally downregulating expression of the junctional protein, claudin 5 (ref. 55). Under these conditions, loss of VWF had a deleterious effect on neurological function and survival. We have shown that VWF expression in the blood–brain barrier is a dynamic mosaic, suggesting that this pattern may have an adaptive role in brain homoeostasis. VWF has also been shown to negatively regulate angiogenesis^53^ by attenuating vascular endothelial growth factor signalling and Ang2 release^54^. Indeed, our data raise the interesting possibility that the noise-sensitive VWF bistable switch in the heart results in transient hotspots of Ang2 release, which in turn may be responsible for vessel remodelling. This hypothesis requires further testing.

Our study has two important limitations. First, we used a knockout model in which *Vwf* has been deleted from the entire mouse. It is thus possible that the localized phenotype in the heart microvasculature is not due to the absence of VWF mosaicism, but rather to the complete loss of VWF throughout the body. Addressing this requires generating a mouse line in which VWF is selectively removed from heart capillary endothelial cells. An additional limitation is our inability to discriminate between the importance of static and dynamic mosaicism. Perhaps what matters most for endothelial health is the mosaic pattern *per se*, and not the dynamic toggling between ON and OFF states. This question could only be addressed by designing a mouse in which VWF expression in the heart is converted from a dynamic to a static mosaic.

These various limitations notwithstanding, our data are the first to support the existence of noise-induced stochastic phenotype switching in adult mammals. Our findings point to biological noise as a new source of phenotypic diversity in the healthy endothelium, which in the case of VWF is exploited by some, but not all, vascular beds. The observation that other genes, including ESM1 and ephrin-B2, also demonstrate dynamic mosaicism *in vitro* raises the possibility that this phenomenon is not unique to VWF but may represent a general, previously unappreciated endothelial cell behaviour.

The traditional motivation for studying both the scope and the molecular basis of endothelial heterogeneity is to design vascular bed-specific therapies. The implicit goal is to inhibit (that is, turn OFF) or accentuate (that is, turn ON) site-specific expression/activity of certain proteins to modulate local endothelial cell behaviour. However, in so far as noise-induced transitions in phenotype are adaptive, therapies aimed towards nudging cells into a single state may be ill advised. The optimal treatment may be one that restores the flexibility to generate mosaic heterogeneity, and thus preserve homoeostatic bet hedging.

## Methods

### Statistics

Two-sided *T*-test was performed in all significance tests, except the clonality assessment of DNA methylation patterns (Supplementary Note 7).

### Approval for experiments involving animals

All experiments involving the generation, manipulation and analysis of mice were carried out in compliance with ethical regulations and were approved by the Institutional Animal Care and Use Committee at the Beth Israel Deaconess Medical Center (BIDMC).

### Generation of targeting constructs

To generate the vWF^LacZ/+^ construct^9^, RPCI-24 mouse genomic BAC clones 365D10 and 211J14 in *Escherichia coli* EL250 cells were grown under chloramphenicol selection, heart-shocked to induce recombination activities and electroporated with the LacZ-pA-fritted neo^r^ cassette from pBXZneoB with ligated homology arms to direct its insertion between the end of intron 1 and nucleotide 4253 of intron 3. The scheme used to capture the native locus in the construction of pVWMDTZ1 was used to capture this targeted locus. To generate the vWF^Cre/+^ targeting construct (pVWKOCKI), recombination proficient cells were co-electroporated with pVWMDTZ1 and a Cre-pA-fritted neo^r^ cassette with ligated homology arms to direct its insertion between the end of exon 1 and nucleotide 4253 of intron 3. The cassette was constructed in three steps. First, a pair of complementary oligos was inserted between blunted *Sph*I and *Bam*HI sites of pUC18 to introduce a flippase recognition target (FRT) site preceded by sites for *Bbs*I and *Pml*I and followed by another *Bbs*I site oriented opposite the first. The *Pml*I site and the *Xba*I site in FRT are unique. Next, a nuclear-localized Cre recombinase coding sequence and human β-actin polyadenylation sequence amplified from pML78 was inserted into the *Pml*I site. Finally, an *Xba*I restriction product containing the neo resistance cassette from pNEOREV was inserted into the *Xba*I site in the orientation that preserved the flanking FRT sites. The orientation of Cre recombinase in the preceding step was chosen to be parallel to neo resistance in the ultimate product. Digestion of this construct with *Bbs*I (after exhaustive *Hind*III digestion to linearize it) released the Cre-pA-fritted neo^r^ cassette as two fragments due to an overlooked internal *Bbs*I site. Each homology arm was amplified with one primer that introduced a *Bsm*BI site and sequence that on digestion complemented the appropriate *Bbs*I digested end. The homology arms and the two cassette fragments were ligated and employed in the above recombination reaction. To generate the vWF^CreERT2/+^ targeting construct, recombination proficient cells were co-electroporated with pVWMDTZ1 and a tamoxifen-inducible Cre-pA-fritted neo^r^ cassette with ligated homology arms to direct its insertion between the end of exon 1 and nucleotide 4253 of intron 3. Construction of the cassette employed the initial construct from preparation of the pVWKOCKI cassette, but in this case the next step was insertion of the neo resistance cassette from pNEOREV. A 2,348-bp amplicon from pCAG-CreERT2 (Addgene ID 14797) containing coding sequence and polyadenylation signal was inserted into the *Pml*I site. Digestion of this construct with *Bbs*I (after exhaustive *Eco*RI digestion to linearize it) released the inducible Cre-pA-fritted neo^r^ cassette. This was ligated with the same homology arms used in the construction of pVWKOCKI.

### Generation of gene-targeted mice

To generate vWF^LacZ^, vWF^Cre/+^ and vWF^CreERT2/+^ mice, W4/129S embryonic stem cells (ES cells) (Taconic Biotechnology) were prepared and transfected with the constructs described above, placed under G418 selection (Invitrogen) for 6–7 days. Homologous recombination in G418-resistant ES cells was confirmed by Southern blot of genomic DNA and specifically designed probes. Positive clones were amplified and submitted to the BIDMC transgenic facility for injection into blastocysts. Tie2^Cre/+^ mice were obtained from Jackson Research Laboratories. To generate vWF–Cre–ROSA26, vWF–CreERT2–ROSA26, or Tie2–Cre–ROSA26 mice, vWF^Cre/+^, vWF^CreERT2/+^ and Tie2^Cre/+^ mice were crossed with ROSA26 mice (Jackson Research Laboratories). Each of these lines has been backcrossed to C57/BL6 for at least nine generations. Unless otherwise stated, all experiments involved male mice between the ages of 6 and 8 weeks.

### LacZ staining and vWF immunohistochemistry

Tissues were collected and fixed in fixative solution containing 270 μl of formaldehyde and 80 μl of gluteraldehyde in 10 ml PBS for 30 min at room temperature. The tissues were washed three times in PBS, and then either incubated with X-gal overnight for whole-mount staining, or embedded in was optimal cutting temperature compound (OCT), sectioned, fixed a second time with fixative solution for 10 min and incubated overnight with X-gal. For immunohistochemistry, slides were fixed in acetone at −20 °C for 2 min, rinsed three times in PBS, and incubated in Dako Ready-to-use Protein Block (Dako Cat. No. X0909) for 10 min at room temperature. Slides were then incubated with primary antibody (polyclonal anti-human VWF, Dako Cat. No. A0082) 1:100 at 4 °C overnight. The slides were then rinsed three times in PBS, blocked with peroxidase (1 ml H_2_O_2_ in 150 ml methanol) for 10 min at room temperature and washed again before incubating with secondary antibody (biotinylated goat anti-rabbit, Dako Cat. No. E0432). After washing in PBS, the slides were incubated with tertiary antibody (Vector Labs ABC Elite PK-6100) and then processed for AEC staining. Finally, the slides were counterstained with Hematoxylin and saturated Lithium Carbonate Solution and sealed with cover slips in aqueous mounting medium.

### Immunofluorescent co-staining of tissue sections

Frozen sections of organs were fixed in cold acetone for 5 min at −20 °C, rinsed in PBS and blocked in blocking solution (5% fish gelatin and 10% donkey and 10% goat sera) for 1 h at room temperature and then incubated with primary antibodies (chicken polyclonal anti-LacZ (Abcam Cat. No. ab9361) diluted at 1:100 and rat anti-mouse CD31 (BD Pharmingen Cat. No. 550274) diluted at 1:50) at 4 °C overnight. Next, slides were washed with high-salt PBS and incubated with secondary antibodies (Alexa Fluor 546 or 488 (Invitrogen), diluted at 1:1,000) for 1 h at room temperature. Following high-salt PBS washes, slides were mounted in 4,6-diamidino-2-phenylindole containing mounting media and analysed using WaveFx Confocal-Spinning Disk microscope and Volacity software.

### Immunofluorescent co-staining of en face aorta

Aortas were fixed in 4% paraformaldehyde (PFA), washed in PBS, and blocked with TNB Blocking Buffer (TNBT) (0.1 M Tris-HCl, 0.15 M NaCl, 0.2% triton-X-100, 0.5% blocking, Perlin Elmer). Aortas were incubated in Image-iT FX signal enhancer (Invitrogen) at room temperature for 30 min and assayed using primary antibodies (rabbit monoclonal vWF (Dako Cat. No. A0082) diluted at 1:1,000, Armenian hamster polyclonal CD31 (Millipore Cat. No. MAB1398Z) diluted at 1:100, and rabbit polyclonal β-galactosidase (MP Biomedicals Cat. No. 08559761) diluted at 1:50) diluted in TNBT at 4 °C overnight. Next, aortas were washed with PBS and incubated with secondary antibodies (Alexa Fluor 594 (Invitrogen) and Alexa Fluor 488 (Jackson ImmunoResearch) diluted at 1:200) for 2 h at room temperature. For whole-mount LacZ and CD31 aorta co-staining, aortas were stained for LacZ first, and then immunostained for CD31 as described above.

### Cell culture

NIH3T3 (a murine fibroblast cell line; ATCC CRL-1658) and HepG2 (an hepatocyte cell line; Sigma-Aldrich, Cat. No. 85011430) were grown in DMEM supplemented with 10% FCS (Hyclone). All primary human endothelial cells were obtained from Lonza and grown in EBM-2 (EC Basal Medium-2) supplemented with EGM-MV SingleQuots (Lonza). HCVSMC and HAoVSMC were also obtained from Lonza and grown in SmBM Basal Medium supplemented with SmGM-2 SingleQuot (Lonza). To isolate primary mouse lung and heart endothelial cells, mouse tissue was harvested on ice, minced and digested in 0.2% (w/v) type I collagenase (Worthington) for 45 min at 37 °C, passed over a 70 micron filter, and spun down at 500G without brake (4 °C). The resulting cell pellet was resuspended in bead wash (PBS +0.1% albumin and antibiotics), and then incubated for 15 min at room temperature with magnetic beads (Dynabeads, Invitrogen) that were precoated with anti-mouse CD31 antibody (BD Biosciences, Cat. No. 553370). After six washes, the cells were harvested for T0 assays, or plated on gelatin-treated tissue culture plates (0.1% gelatin, Millipore) and grown to confluence in DMEM with 20% FBS, amino acids, glutamax, antibiotics and BT203 endothelial mitogen (Biomedical Technology, Inc.). A second beading was performed with magnetic beads coated with anti-mouse ICAM2 antibody (BD Biosciences, Cat. No. 553326) and plated on gelatin-treated plates. Primary mouse hepatocytes were purchased from BD Biosciences. To obtain mouse sinusoidal endothelial cells, freshly harvested livers were washed with Dulbecco's PBS (DPBS) and minced. Liver material was then suspended in 0.2% (w/v) type I collagenase (Worthington) in DPBS and incubated in an overhead shaker at 37 °C for 45 min. Liver cells were further dissociated by repeated suction through a 14-gauge needle and filtered through a 70-μm cell strainer (BD Biosciences). The cell suspension was centrifuged for 1 min at 500G without brake (4C). The hepatocyte-containing pellet was resuspended in DPBS and mixed with OptiPre Density Gradient Medium (Sigma) at a ratio of 3:1, layered with DPBS and centrifuged for 15 min at 1500G without brake (4 °C). Cells in the interphase were collected, washed in DPBS, centrifuged for 10 min at 500G and resuspended in bead wash buffer (PBS with antibiotic-antimycotics and BSA; all from Mediatech, Herndon, VA). Next, Dynabeads sheep anti-rat IgG (Invitrogen) conjugated with rat anti-mouse CD31 monoclonal antibody (BD Biosciences Cat. No. 558736) were added and the mixture was incubated in an overhead shaker for 15 min at room temperature. Cells were repeatedly washed with bead wash buffer using a magnetic cell sorter before harvesting in DNA isolation buffer.

### Fluorescent *in situ* hybridization

FISH was performed using QuantiGene ViewRNA ISH Cell Assay Kit (Affymetrix), following the manufacturer's instructions with minor modifications. Briefly, cells were plated on fibronectin-coated cover slips, fixed in 4% formaldehyde for 10 min, permeabilized with detergent solution for 5 min and treated with protease for 10 min at room temperature and incubated with gene-specific probes for 3 h at 40 °C (probes, hvWF: nm_000552, 2460–3950; hESM1: nm_007036, 542–2049; hROBO4: nm_019055, 713–1896; hVE-CAD: nm_001795, 284–1406; hEFNB2: nm_004093, 54–979; hERG: nm_004449, 107–1179; mvWF: nm_011708, 3702–4782. LacZ: U46489, 519–1433), followed by incubation with preamplifier Mix, Amplifier Mix and Label probe Mix, sequentially. The slides were rinsed three times in PBS and mounted with cover slips using VectaShield mounting medium containing 4,6-diamidino-2-phenylindole for nuclear imaging. Images were collected using Zeiss LSM 510 laser-scanning confocal microscope with a 40 × oil-immersion objective.

### Quantitative real-time PCR

Total RNA was isolated using the RNAeasy kit (Qiagen). Single-stranded complementary DNA (cDNA) was synthesized from total RNA using the RNA-to-cDNA kit (Applied Biosystems). SYBR Green I-based real-time PCR was carried out on an Opticon Monitor. For each run, 18 s ribosomal RNA were used to normalize the amount of cDNA. The sequences of the primers used in this study are listed in Supplementary Table 1.

### Arterial and venous flow experiments

HPAEC were plated onto 0.1% gelatin-coated shear plates at a cell density of 72,000 cm^−2^. The samples were exposed for 24 h to arterial or venous shear stress waveforms derived from the human abdominal aorta or saphenous vein, respectively using a dynamic flow system^65^. Average mRNA expression and DNA methylation from these cells was used in Fig. 5b, as the medium-high VWF expression in these endothelial cells helped cover the range between high-VWF HUVECs and low-VWF aortic endothelial cells, leading to a more robust fit (24 h exposure to venous versus arterial had no measurable effect on VWF expression or DNA methylation).

### Generation of luciferase constructs

pvWF-Met1 was constructed on the basis of pvWF4-LUC using the PCR amplicon from a 357 bp Integrated DNA technologies (IDT) gBlock synthesis in which the three CpG sites in exon 1 were changed to TpG. pvWF-Met2/3/4 were generated on the basis of pvWF2 (vWF-WT). The *VWF* promoter CpG sites at −790, −769, −478 and −422 were either all mutated to TpG (vWF-Met4), or the upper pair were mutated alone (vWF-Met2), as were the lower pair (vWF-Met3). All of these sites are contained within an 869-bp *Bst*XI restriction fragment of pvWF2-LUC3. To subclone this fragment, pUC18 was modified by inserting annealed oligos that contained the terminal *Bst*I sites and cohesive ends for *Sph*I and *Sac*I between the vector *Sph*I and *Sac*I sites. Insertion of the *Bst*XI fragment into this modified pUC18 yielded pVWHp-BSTX. The insert contains a unique *Kpn*I site (at −622) and is flanked by unique vector sites for *Hind*III and *Sac*I. The regions between *Hind*III and *Kpn*I and between *Kpn*I and *Sac*I were within the range of gBlock synthesis by integrated DNA technologies. When these were submitted (with the pertinent mutations), the first was rejected. This region contains the (GpT)_20_ repeat as well as a run of six G's on the anti-sense strand. The second was synthesized and inserted between *Kpn*I and *Sac*I of pVWHp-BSTX to yield pVWHpWm-BX. The upper pair of CpG sites were mutated employing a 45-mer sense oligo centred on them. The complementary strand could not be synthesized as it contained the run of six G's. Instead, a complementary 18-mer was annealed with the 45-mer and extended with Klenow. This duplex, combined with pVWHp-BSTX vector in a KOD polymerase reaction, was subjected to 18 thermal cycles, digested with *Dpn*I to remove wild-type sequences and transformed. This produced pVWHpmW-BX. The *Sac*I to *Kpn*I major fragment from this, combined with the *Kpn*I to *Sac*I small fragment from pVWHpWm-BX, produced pVWHpmm-BX. The 6,543-bp *Bst*XI fragment from digestion of pVW2-LUC3 and the 879- and 134-bp *Bst*XI fragments from digestion of a gel-purified 1,514-bp *Xba*I to *Kpn*I fragment of pvWF2-LUC3 were combined in ligation reactions with the 869-bp *Bs*tXI fragments from the preceding three constructs to re-constitute pvWF2-LUC3 carrying the mutations.

### Methylation of promoter/reporter constructs

The human *VWF* promoter/luciferase reporter constructs were *in vitro* methylated by M.SssI CpG methyltransferase (New England Biolabs) according to the manufacturer's recommendations. Mock methylation reactions did not contain any methylase. Methylated and mock-methylated constructs were phenol/chloroform purified and precipitated in ethanol before transient transfection experiments.

### Luciferase assay

HUVEC were plated in a 12-well plate the day before transfection. Methylated or mock-methylated *VWF* promoter pGL3 constructs were transfected as indicated along with Renilla (Promega) using Dharmafect I (Dharmacon). After 24 h of incubation, the cells were lysed in 200 μl of Cell Culture Lysis Reagents (Promega) and analysed for Luciferase activity by using Luciferase Assay System (Promega) with AutoLumat LB953 (EG&G Berthold).

### Flow cytometry and FACS

Cells were trypsinized, washed twice in PBS (without Ca^2+^ and Mg^2+^), incubated in a non-enzymatic cell dissociation solution (Sigma) for 10 min at room temperature followed by permeabilization solution for 5 min and three washes in PBS. The cells were then labelled with anti-vWF antibody (Dako Cat. No. A0082) at a dilution of 1:1,000, followed by incubation with secondary antibody of FITC (Jackson Research Laboratories). The labelled cells were washed with PBS. Flow cytometry runs and FACS sorting were performed on the FX5000 Flow Cytometer/FACS sorter at the BIDMC Flow Cytometry Facility, using the CXP analysis software. For FACS sorting, the highest and lowest 10% cells were collected and designated as VWF high and low, respectively.

### Sodium bisulfite genomic sequencing

Given that the nascent strand of replicating DNA is hemimethylated immediately following DNA replication, only quiescent post-confluent primary cells were utilized for DNA isolation. Briefly, 2 μg of mechanically sheared genomic DNA was denatured with 0.3 M NaOH for 15 min at 37 °C, and then incubated with 3.1 M sodium bisulfite and 0.5 mM hydroquinone at 55 °C for 16 h in the absence of light. Free bisulfite was removed using UltraClean 15 DNA Purification kit (MO BIO Laboratories), followed by alkali desulfonation with 0.2 M NaOH for 10 min at 20 °C. Sample DNA was neutralized with NH_4_OAc, precipitated and resuspended in nuclease-free water. 25 ng of the bisulfite-treated DNA was subjected to 35 cycles of PCR amplification in a volume of 50 μl using the outer primers. 2 μl of the PCR product was used as template for another 35 cycles of nested PCR amplification in a volume of 50 μl using the nested (inner) primers. All PCR primers were specifically designed to the sodium bisulfite-modified sense strand. The final PCR products were subcloned using TOPO TA cloning kit (Invitrogen) to yield individual strands, followed by sequencing. For each cell type at least 15 randomly chosen subclones were sequenced. Primers used for human *VWF* upstream region were hvWF-upstream-outer-F, hvWF-upstream-outer-R, hvWF-upstream-inner-F and hvWF-upstream-inner-R; primers used for human *VWF* core promoter region were hvWF-core-outer-F, hvWF-core-outer-R, hvWF-core-inner-F and hvWF-core-inner-R; primers used for mouse *Vwf* proximal promoter region were mvWF-prox-outer-F, mvWF-prox-outer-R, mvWF-prox-inner-F and mvWF-prox-inner-R; primers used for mouse *Vwf* exon1 region were mvWF-exon-outer-F, mvWF-exon-outer-R, mvWF-exon-inner-F and mvWF-exon-inner-R. The sequences of these primers are listed in Supplementary Table 1.

### Correlation between average mRNA level and DNA methylation

To quantify the relationship between average DNA methylation and mRNA expression, we used a log-linear fit on all assayed cell types and endothelial cell subtypes (that is, log(mRNA)∼average DNA methylation). Cell types used for plot: HUVEC; HPAEC—two separate preparations, venous flow, arterial flow; HMVECs—two separate preparations; human aortic endothelial cells, HCAVSMCs and hepatocytes (all were primary human cells). The resulting exponential, shown in Fig. 5b, is described by *y*=10^(−6.5±0.7) × *x*+(3.3±0.3)^, with a correlation coefficient of *C*=−0.95.

### ChIP and bisulfite sequencing of ChIP DNA (ChIP-BS-seq)

Chromatin immunoprecipitation (ChIP) was performed on post-confluent primary cells using the ChIP Assay Kit (Millipore) according to the manufacturer's protocol. Sonication yielded soluble chromatin fragments containing DNA ranging in size from 200 to 400 bp. Histone H2A (C terminus) rabbit polyclonal antiserum (Cat. No. 07–146, diluted at 1:360), histone H2B (C terminus) rabbit polyclonal IgG antibody (Cat. No. 07–371, diluted at 1:360), histone H4 (unmodified/pan) rabbit monoclonal IgG antibody (Cat. No. 05–858, diluted at 1:360), polyvalent acetylated histone H3 rabbit polyclonal IgG antibody (K9, K14; Cat. No. 06–599, diluted at 1:360), polyvalent acetyl histone H4 rabbit polyclonal antiserum (K5, K8, K12, K16; Cat. No. 06–866, diluted at 1:360) and dimethyl histone H3 K4 rabbit antiserum (Cat. No. 07–030, diluted at 1:360) were from Millipore (Temecula). Histone H3 (C terminus) rabbit polyclonal antibody (Cat. No. ab1791, diluted at 1:360), dimethyl histone H3 K9 rabbit polyclonal antibody (Cat. No. ab1220, diluted at 1:288), and tri-methyl histone H3 K9 rabbit polyclonal antibody (Cat. No. ab8898, diluted at 1:360) were from Abcam. RNA polymerase II NH_2_-terminal (N-20) rabbit polyclonal antibody (Cat. No. sc-899 X diluted at 1:720) was from Santa Cruz Biotechnology (Santa Cruz). The immunoprecipitated DNA sample was used in real-time PCR quantification (Applied Biosystems). The primers for human *VWF* loci reside in the same 5′ regulatory regions analysed by the bisulfite method. Primers used for human *VWF* −829 to −780 region were hVWF −829/−780 F and hVWF −829/−780R; primers used for human *VWF* −480 to −380 region were hVWF −480/−380 F and hVWF −480/−380R; primers used for human *VWF* +124 to +233 region were hVWF 124/233 F and hVWF 124/233R. The amount of template present was calculated relative to a standard curve. IP DNA was calculated by first subtracting the amount of sequence present in the no antibody background control or IgG control from the amount present in the IP DNA, and then divided by the amount of sequence in the diluted input material. Primers used for bisulfite sequencing of ChIP DNA were hvWF-upstream-ChIP-outer-F, hvWF-upstream-ChIP-outer-R, hvWF-upstream-ChIP-inner-F and hvWF-upstream-ChIP-inner-R; hvWF-core-ChIP-outer-F, hvWF-core-ChIP-outer R, hvWF-core-ChIP-inner-F and hvWF-core-ChIP-inner-R. The sequences of these primers are listed in Supplementary Table 1.

### 5-Aza treatment of cultured cells

HUVEC and HAoVSMC were seeded at a density of 3–4 × 10^5^ cells on 100 mm dishes. Exponentially growing cells were treated with 5-Aza (Sigma-Aldrich) every 24 h for 7 days. Maximally tolerable dose of 5-Aza was used for HUVEC (5 μM) and HAoVSMC (0.5 μM). Medium was changed on the fourth and sixth days of treatment. On the eighth day, cells were harvested for genomic DNA and total cellular RNA.

### Hairpin bisulfite sequencing

Given that the nascent strand of replicating DNA is hemimethylated immediately following DNA replication, only quiescent post-confluent primary cells were utilized for DNA isolation. Briefly, 10 μg of genomic DNA was cleaved with *Sca*I (FastDigest *Sca*I restriction enzyme FD0434; Thermo Scientific) to generate a 859-bp fragment of the 5′ flanking region of human *VWF*. After heat inactivation of *Sca*I and buffer exchange, the gene fragment was further cleaved with *Tsp*45I (*Tsp*45I restriction enzyme R0583, New England Biolabs) close to the *VWF* promoter region of interest. QIAEX II DNA cleanup kit (20021, Qiagen) was used to purify and concentrate DNA fragments from *Tsp*45I, which cannot be heat inactivated. Hairpin linker (5′-P-GTGACAGCGATGCDDDDDDDGCATCGCT-3′) was ligated to the staggered ends of the *Tsp*45I-cleaved genomic DNA, covalently joining the two complementary strands of an individual DNA molecule. Sodium bisulfite conversion was then carried out using Imprint DNA modification kit (MOD50, Sigma-Aldrich) according to manufacturer's protocol, but with the addition of a heat-denaturing step (99 °C for 2 min) during the 90 min of bisulfite conversion (65 °C) to denature the hairpin-linked DNA. Bisulfite-treated DNA was subjected to 40 cycles of PCR amplification in a volume of 50 μl using *VWF*-specific forward primer hVWF-hairpin-F and reverse primer hVWF-hairpin-R; annealing temperature=47.5 °C. 10 μl of the PCR product (586 bp) was used as template for another 40 cycles of nested PCR amplification in a volume of 50 μl using *VWF* nested forward primer hVWF-hairpin-nested-F and reverse primer hVWF-hairpin-nested-F; annealing temperature=46.4 °C. The final PCR product (550 bp), containing CpG dyads at +119, +179 and +227 of human *VWF*, was subcloned using TOPO TA cloning kit to yield individual DNA molecules, followed by sequencing. Randomly variable barcode encoded in the loop of the hairpin linker was used to identify and remove redundant methylation patterns arising from PCR amplification of limited amounts of DNA template^46^.

### Transmission electron microscopy

Plastic sections (1 μm) were stained with alkaline Giemsa stain for study by light microscopy, and 70- to 80-nm thin sections were examined by electron microscopy with Philips 300, 400 and CM10 electron microscopes (Philips, Eindhoven, The Netherlands) by three different microscopists.

### PV loop, echocardiography and aortic banding

For pressure–volume loop, pressure–volume parameters were measured under isoflurane (2%) inhalant anaesthesia using a 1.4-Fr microtip pressure–volume catheter (Scisense), inserted into the right common carotid artery and advanced into the left ventricle. Data were recorded using a PowerLab system (AD Instruments). Beat-by-beat pressure–volume parameters including stroke work, cardiac output, preload, afterload and contractility were measured and analysed using CardioSoft Pro software (CardioSoft). For echocardiography, the chest hair was removed and two-dimensional images were visualized using a Vevo 2100 system. Non-invasive M-mode imaging could accurately measure cardiac function, left ventricular wall thickness and chamber dimensions. For surgical aortic banding, mice under anaesthesia were placed supine on a heating pad. A horizontal skin incision of 0.5–1.0 cm in length was made at the level of the suprasternal notch and aortic arch was exposed. A wire with a snare on the end was passed under the aorta between the origin of the right innominate and the left common carotid arteries. A 6–0 silk suture was snared with the wire and pulled back around the aorta. A bent 27-gauge needle was then placed next to the aortic arch and the suture was tied around the needle and the aorta. Following ligation, the needle was quickly removed. The skin was closed and mice were allowed to recover. The sham procedure was identical except that the aorta was not ligated.

### Assaying liver and kidney function

Blood was collected via cardiac puncture and assayed for liver and kidney function using Chem-27 assay commercial services provided by the Diagnostic Lab in the Division of the Comparative Medicine, Massachusetts Institute of Technology.

### Angiopoietin 2 ELISA

was performed using Mouse/Rat Angiopoietin 2 Quantikine ELISA Kit (antibody1:200; R R&D Systems) following the manufacturer's instruction.

## Additional information

**How to cite this article:** Yuan, L. *et al.* A role of stochastic phenotype switching in generating mosaic endothelial cell heterogeneity. *Nat. Commun.* 7:10160 doi: 10.1038/ncomms10160 (2016).

## Supplementary Material

Supplementary Supplementary Figures 1-10, Supplementary Tables 1-3, Supplementary Notes 1-9 and Supplementary References
