# Supplementary material for: A role of stochastic phenotype switching in generating mosaic endothelial cell heterogeneity
Source: Nat Commun. 2016 Jan 8;7:10160. doi: 10.1038/ncomms10160 (PMC5154372; doi:10.1038/ncomms10160)
Supplement: Supplementary — Figures 1-10, Supplementary Tables 1-3, Supplementary Notes 1-9 and Supplementary References [file ncomms10160-s1.pdf]

Supplementary Figures

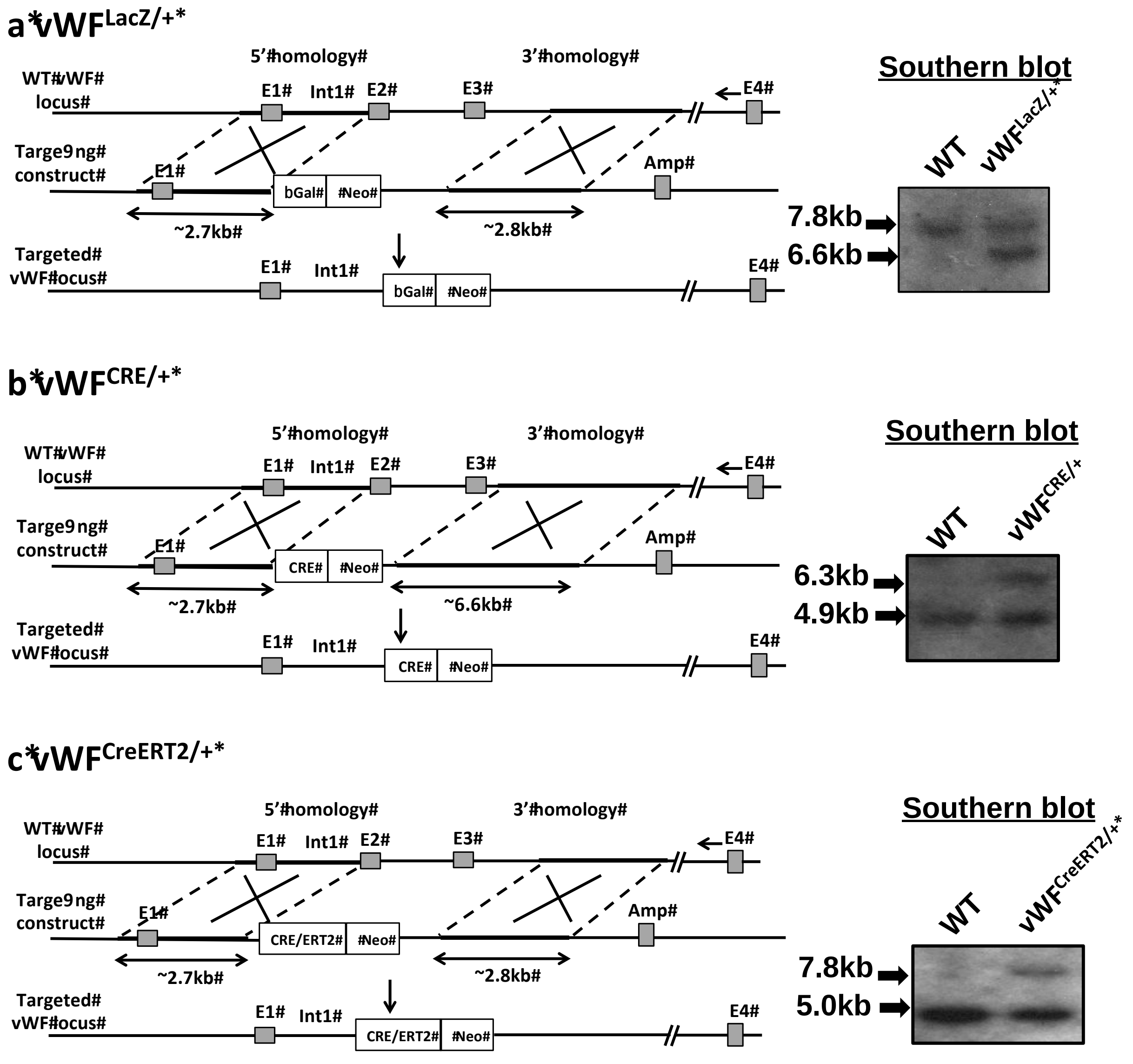

Supplementary Figure 1. Generation of targeting constructs. *Left*, schematics for targeting the *lacZ* reporter gene to the endogenous *Vwf* locus (vWF$^{LacZ/+}$) (**a**), Cre recombinase to the endogenous *Vwf* locus (vWF$^{Cre/+}$) (**b**) and tamoxifen-inducible Cre recombinase to the endogenous *Vwf* locus (vWF$^{CreERT2/+}$) (**c**). *Right*, Southern blots of wild type (WT) (a) and targeted ES cells (b-c). E1, exon 1; E2, exon 2; E3, exon 3; E4, exon 4; Int1, intron 1; β-Gal, LacZ cDNA, Neo, neomycin cassette.

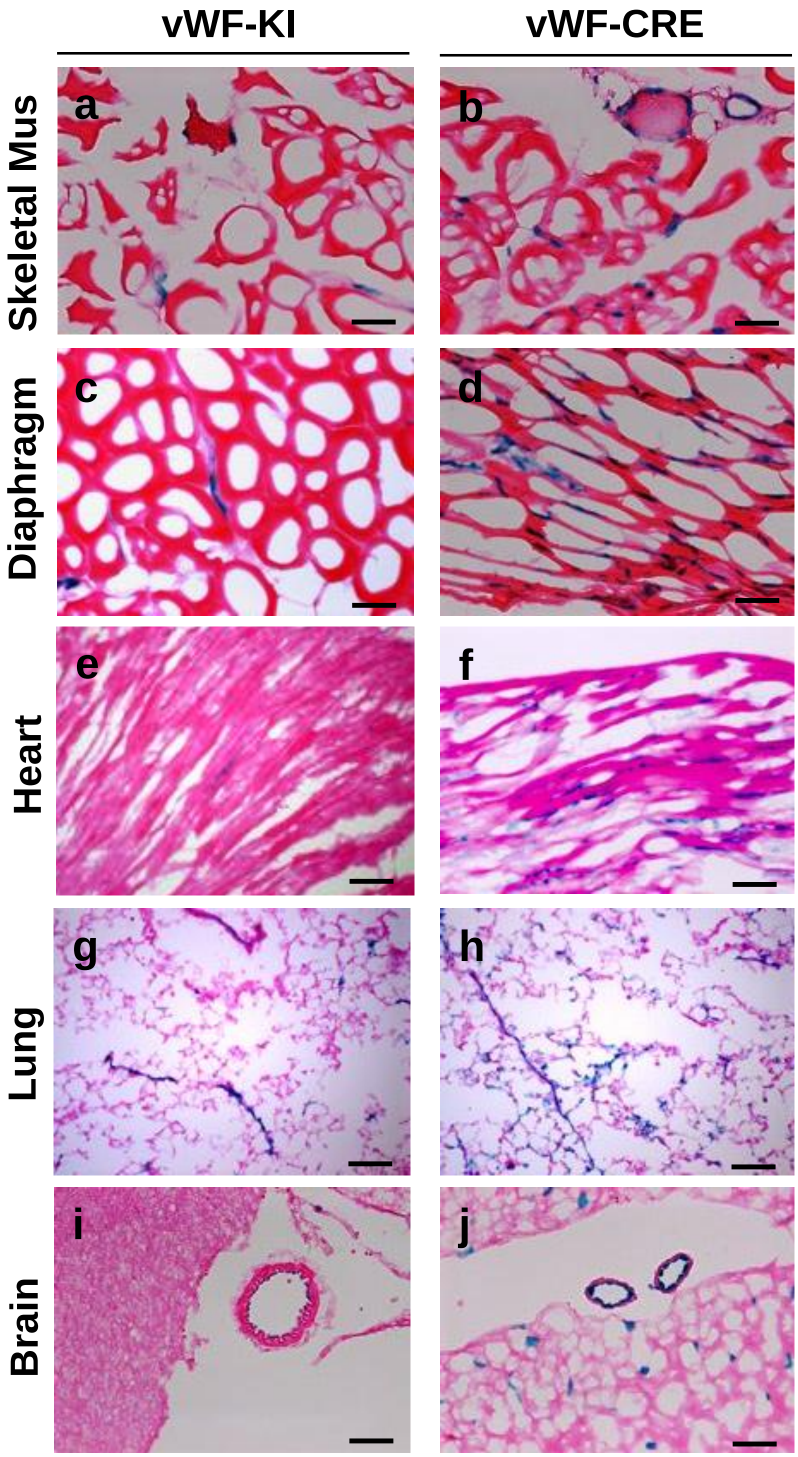

Supplementary Figure 2. Dynamic vWF mosaic heterogeneity in skeletal muscle, heart, lung and brain. Various organs were collected from adult male vWF$^{LacZ/+}$ (vWF-KI) mice and vWF-Cre-ROSA26R (vWF-CRE) mice, cryosectioned and incubated with X-Gal (blue). Comparison of LacZ staining in vWF-KI vs. vWF-CRE capillaries of skeletal muscle (**a, b**), diaphragm (**c, d**), heart (**e, f**), lung (**g, h**) and brain (**i, j**) tissue reveal a dynamical changing vWF mosaic. *n* = 5, with 3 replicates. Scale bar: (a-d) 75µm; (e-j) 150µm.

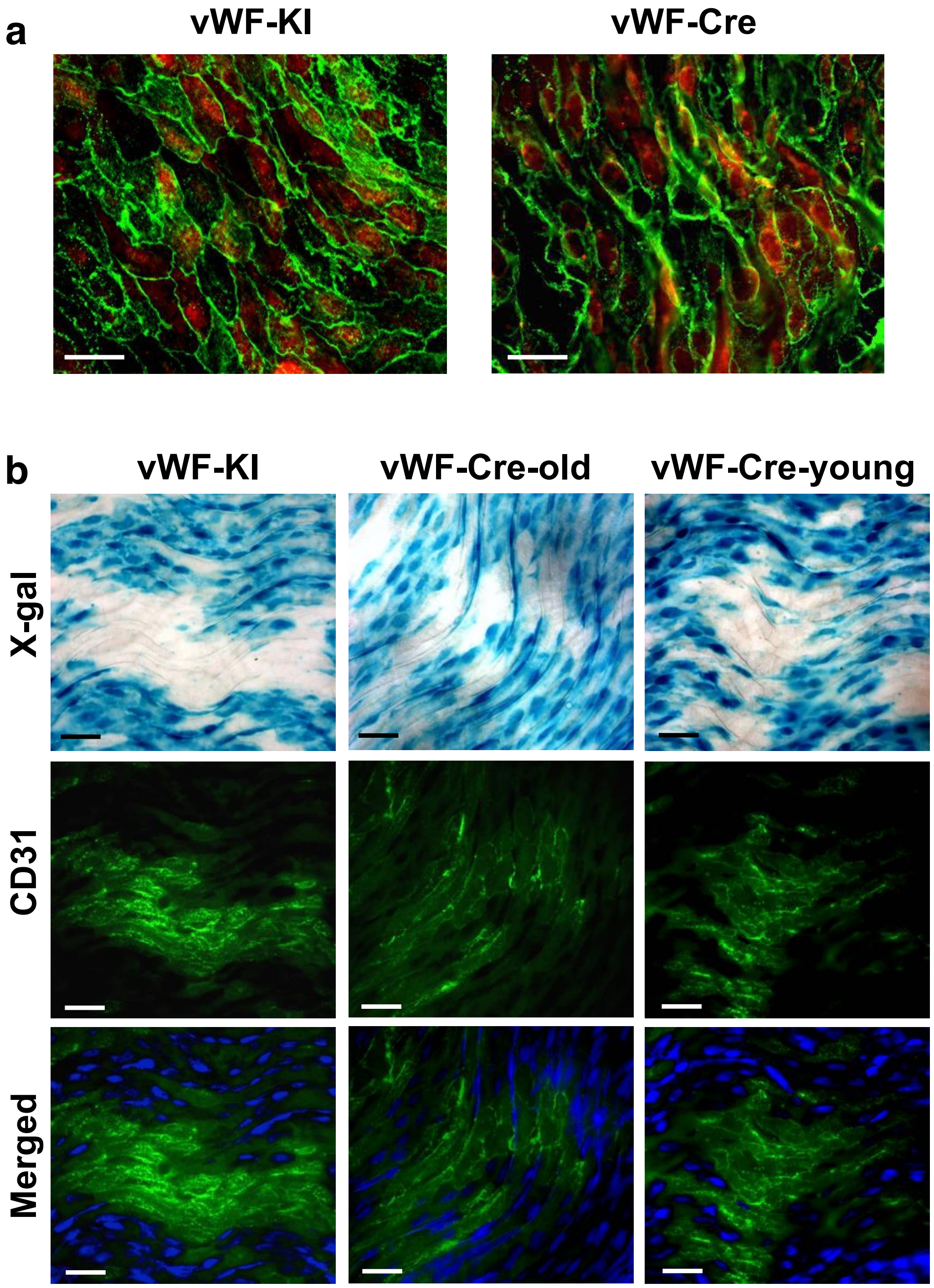

Supplementary Figure 3. vWF demonstrates a static mosaic in the aorta of young and old mice. a. Immunofluorescent co-staining of en face aortas collected from age-matched, 12 month old vWF-KI or vWF-Cre mice and stained for CD31 (green, which outlines the borders between endothelial cells) and LacZ (red). *n* = 3. **b.** En face aortas collected from young (4-week) or old (12-month) vWF-Cre mice, as well as 12-month old vWF-KI mice, stained for LacZ (blue). The tissue was then processed for immunofluorescent CD31 staining (green), which – owing to an artifact of staining - only marked LacZ-negative cells. *n* = 5, with 3 replicates. Scale bar: 75μm.

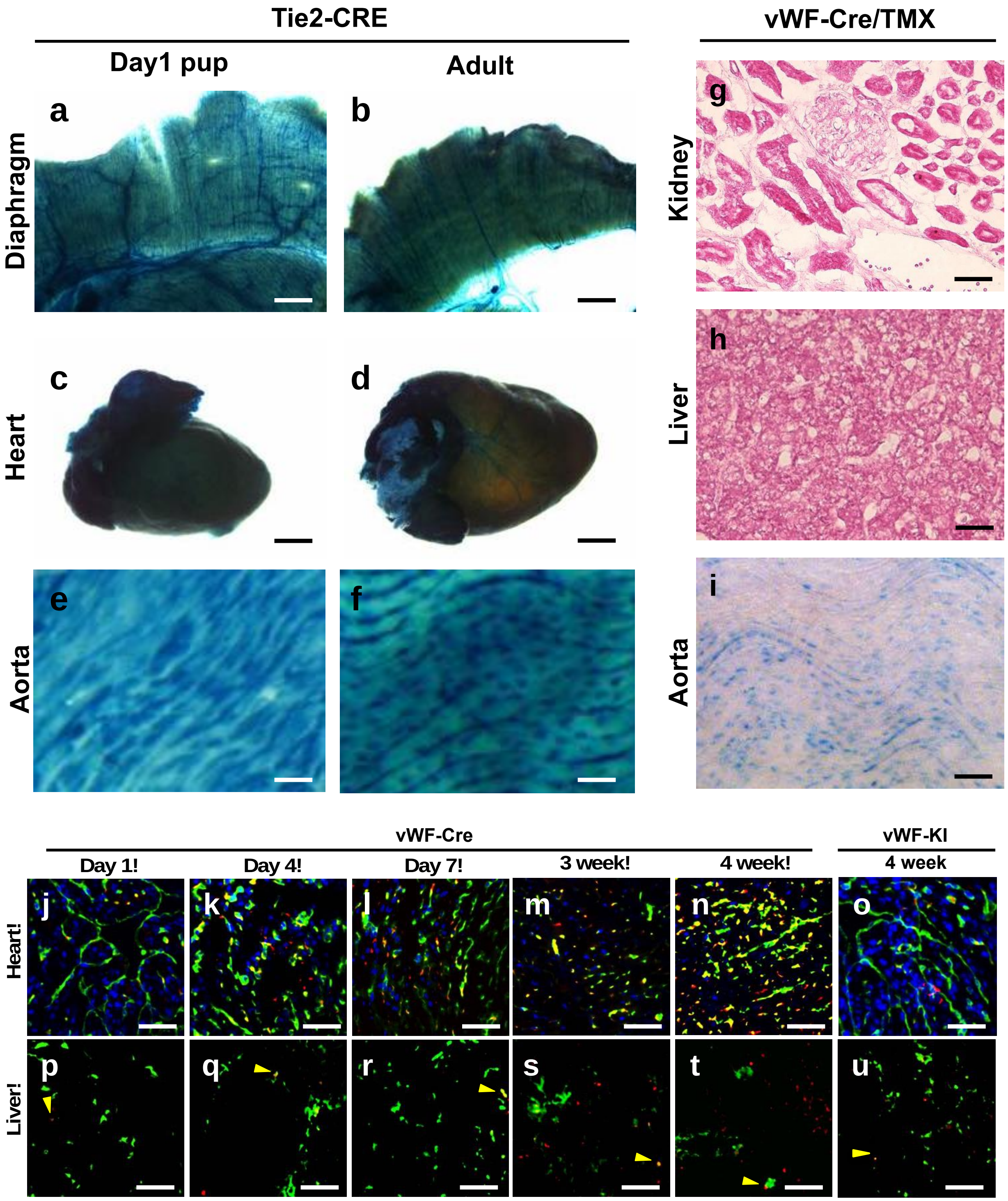

Supplementary Figure 4. LacZ expression in Tie2-Cre-ROSA26R, vWF-Cre/TMX-ROSA26R and vWF-Cre-ROSA26R mice. a-f. A comparison of whole- mount LacZ staining of diaphragm (a-b), heart (c-d) and aorta (e-f) in newborn (day 1, left) and adult (right) Tie2-Cre-ROSA26R (Tie2-CRE) mice shows no difference in saturation between newborn and adult mice. n=5, with 3 replicates. Scale bar: (a,c) 0.75mm; (b,c) 3mm; (e-f) 75µm. **g-i.** LacZ staining of vWF-Cre/TMX liver, kidney and aorta. After vWF-Cre/TMX mice were induced by TMX for 4

weeks, tissues including liver, kidney, and aorta were collected and stained for LacZ (blue). Scale bar: 150μm. j-u. Representative images of vWF-Cre-ROSA26R (vWF-Cre; **j-n**) and $vWF^{LacZ/+}$ (vWF-KI; **o**) heart tissue sections at postnatal days 1, 4, 7, 21 and 28 were immunostained for LacZ (red), CD31 (green) and DAPI (blue), showing time-dependent increase in LacZ/CD31 double-stained ECs (yellow). Representative images of vWF-Cre-ROSA26R (**p-t**) and $vWF^{LacZ/+}$ (**u**) liver tissue sections at postnatal days 1, 4, 7, 21 and 28 were stained for LacZ (red) and CD31 (green), showing very few vWF/CD31 double-positive ECs and no time-dependent increase in LacZ-positive ECs (arrows). *n* = 5, with 3 replicates. Scale bar: 150μm.

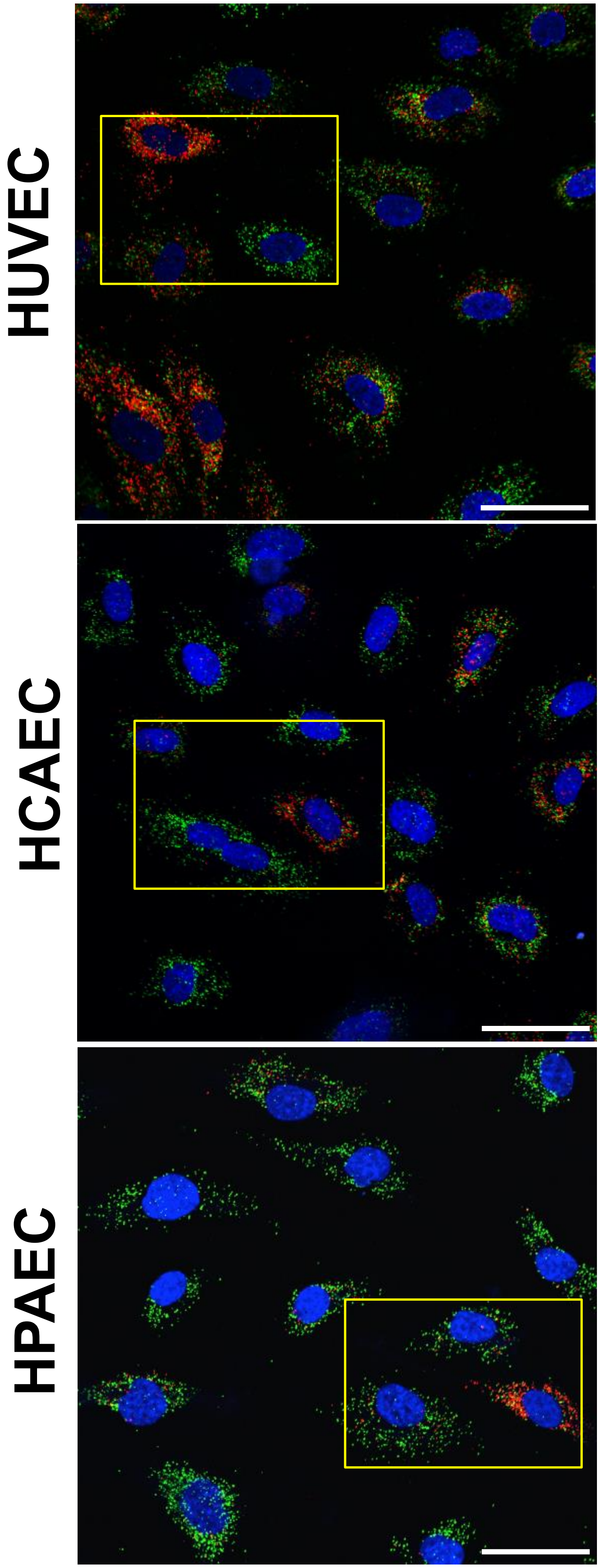

Supplementary Figure 5. Mosaic vWF mRNA expression *in vitro*. FISH for vWF (red) and VE-cadherin (green) in human umbilical vein endothelial cell (HUVEC), human coronary artery endothelial cell (HCAEC) and human pulmonary artery endothelial cell (HPAEC); image shows the larger fields from which the panels of Fig. 4a are magnified (yellow box). *n* =10, with 3 replicates. Scale bar: 40μm.

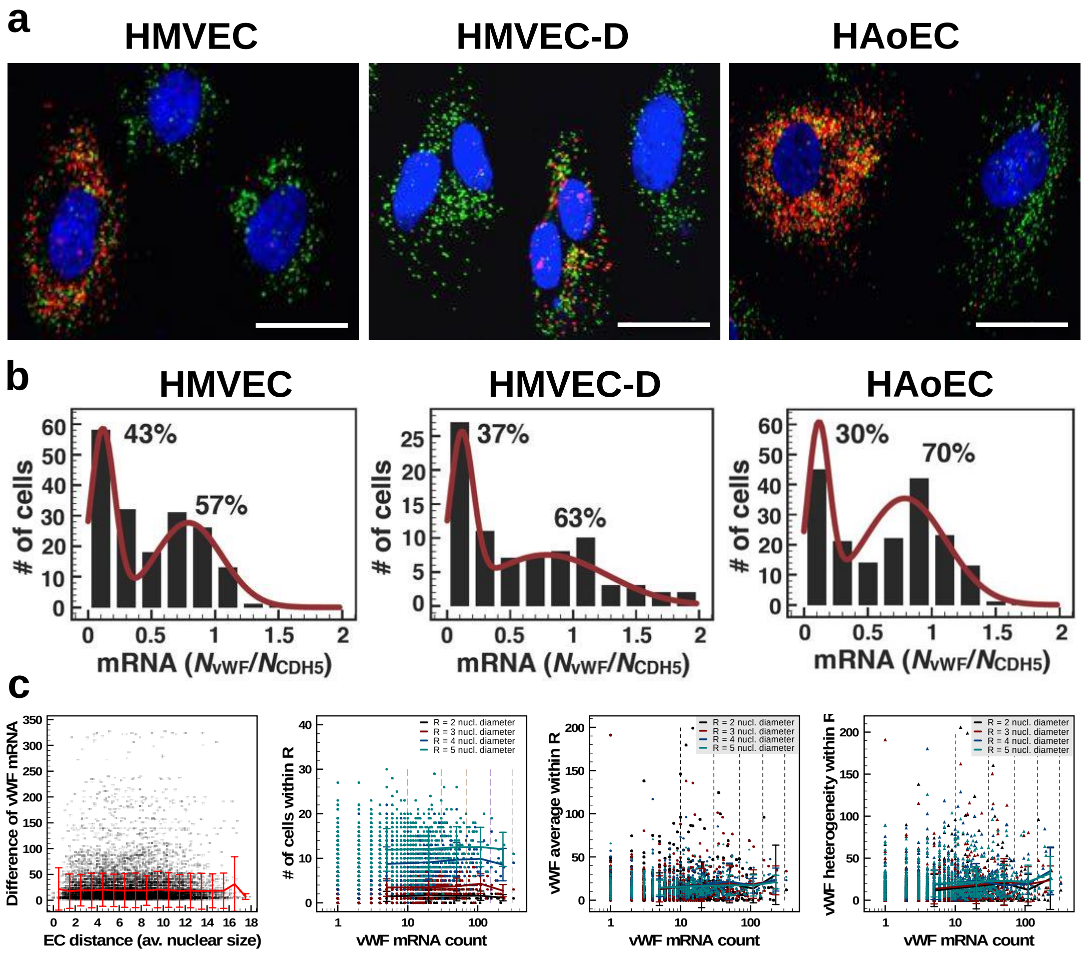

Supplementary Figure 6. Mosaic vWF mRNA expression *in vitro* does not show spatial correlation among EC states. a. FISH staining of vWF (red) and VE-cadherin (green) mRNA (blue: DAPI) in primary human ECs, including cardiac microvascular ECs (HMVEC) (left), dermal microvascular ECs (HMVEC-D) (middle), and aortic ECs (HAoEC) (right). n=10, with 3 replicates. Scale bar: 20µm. **b.** vWF mRNA distribution in populations of HMVEC, HMVEC-D and HAoEC. **c.** *First*, EC pairs at all distances have similarly heterogeneous vWF mRNA expression, as shown by difference in vWF mRNA between pairs of mouse heart ECs as a function of distance (vWF mRNA count was estimated as the sum of vWF and LacZ staining; EC distance was normalized to average nuclear diameter of ~50 ECs). Red line/error bars: averages/standard deviation within 1 nuclear-diameter windows. *Second*, low and high vWF-expressing cells reside in neighborhoods show comparable neighbor density, as indicated by the number of ECs within a radius of 2, 3, 4 and 5 nuclear diameters from an EC as a function of log vWF mRNA in the central cell. Solid lines: averages in windows of increasing size: (10 [1-10], 20 [10-30], 40 [30-70], 80 [70-150] and 160 [150- 310]). *Third* and *fourth*, Low/high vWF-expressing cells have neighborhoods with comparable average vWF expression (*third*) and heterogeneity (*fourth*), as indicated by the average (*third*) and standard deviation (*fourth*) of vWF in ECs within 2, 3, 4 and 5 nuclear diameters from an EC, as a function of log vWF mRNA in the central cell. Solid lines: averages within the above windows.

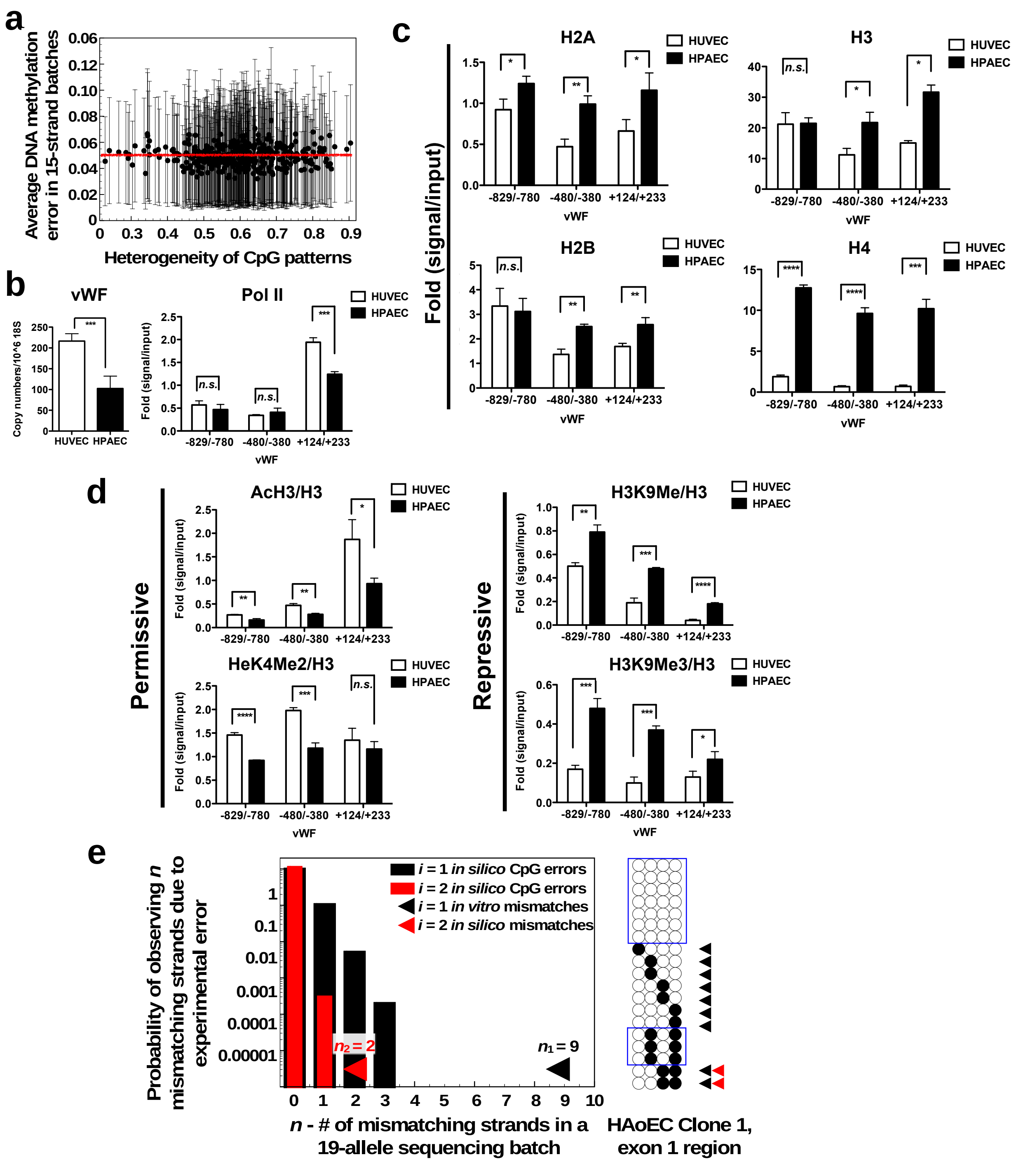

Supplementary Figure 7. Histone modifications and nucleosomal occupancy of the vWF promoter correlates with transcription. a. Average sampling error of the average DNA methylation of a 4-CpG DNA strand in a population, with n = 15 sequenced alleles (estimated by repeated sampling of 400 distinct, heterogeneous *in silico* populations, each harboring all 16 CpG patterns with different weights w_i; heterogeneity of the mix was quantified by its entropy). **b.** vWF RT-PCR (*left*) and polymerase II (Pol II) ChIP of vWF promoter regions (*right*) in HUVEC vs. HPAEC. **c**. Histone occupancy of the vWF promoter (H2A, H2B, H3 and H4 ChIP).

d. ChIP of permissive acetylated histone H3 (AcH3), dimethylated H3K4 (H3K4Me2) and repressive dimethylated H3K9 (H3K9Me2) and trimethylated H3K9 (H3K9Me3) histone modifications at the vWF promoter in HUVEC vs. HPAEC. To account for varying levels of nucleosomal occupancy, ChIP signals were normalized by the H3 occupancy values. b-d. Error bar: standard deviation. Two-sided T-test was performed in all significance tests. n =3, with 3 replicates. * p<0.05; ** p<0.01; *** p<0.001; **** p<0.0001; n.s. p>0.05. **e.** Probability of observing n strands with patterns that do not match the two highest- frequency patterns due to experimental error (*in silico*), compared to *in vitro* results (black/red bars: probability of n strands with 50% mismatching CpGs).

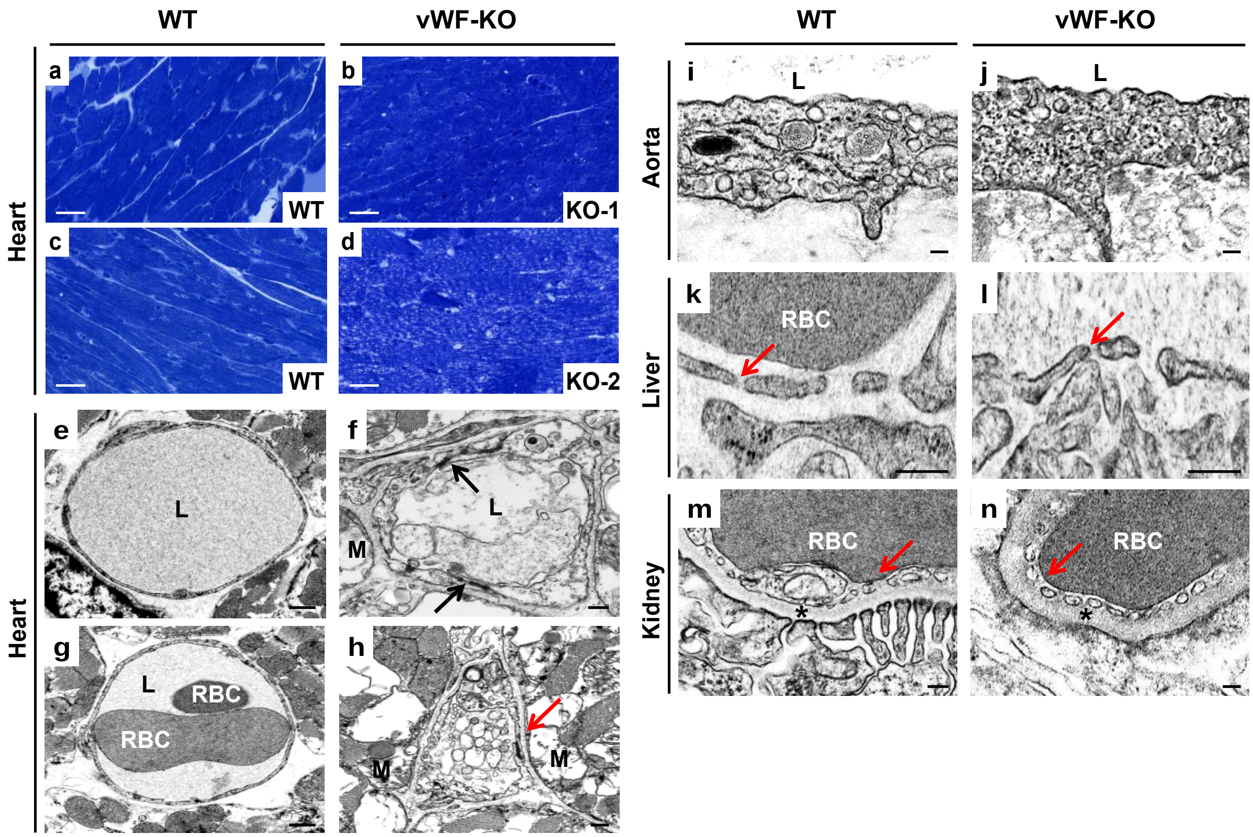

Supplementary Figure 8. Absence of vWF is associated with abnormal cardiac endothelial phenotype. a-d. One-micron Giemsa-stained sections of the heart from wild- type (WT) vs. VWF knockout (KO) mice. Heart capillaries are cut longitudinally in all panels. Note the loss of capillary space in the KO sections, and the frothy appearance of cardiomyocytes in KO-2. Scale bar: 150µm. **e-h.** EM of the wild type heart capillary shows a thin attenuated endothelium. The lumen contains 2 red blood cells (RBC). The heart capillary from the knockout mouse shows electron-lucent endothelial cells with a well-defined lateral border (arrow). The lumen is filled with sloughed membrane bound vesicles. Underlying the endothelium are myocytes containing electron-lucent (injured) mitochondria (M). **i-l.** The aortic endothelium of wild type and knockout mice show abundant caveolae and vesicles. L, lumen. **k-l.** Liver sinusoids in wild type and knockout mice show normal fenestrated endothelium (arrows). **m-n.** The kidney glomerulus of the wild type and knockout mice show normal fenestrated endothelium (arrow), basal lamina (asterisk) and red blood cell (RBC)-filled lumens. Scale bar: (a-d) 500 nm; (e-j) 1 µm. n = 3, with 3 replicates.

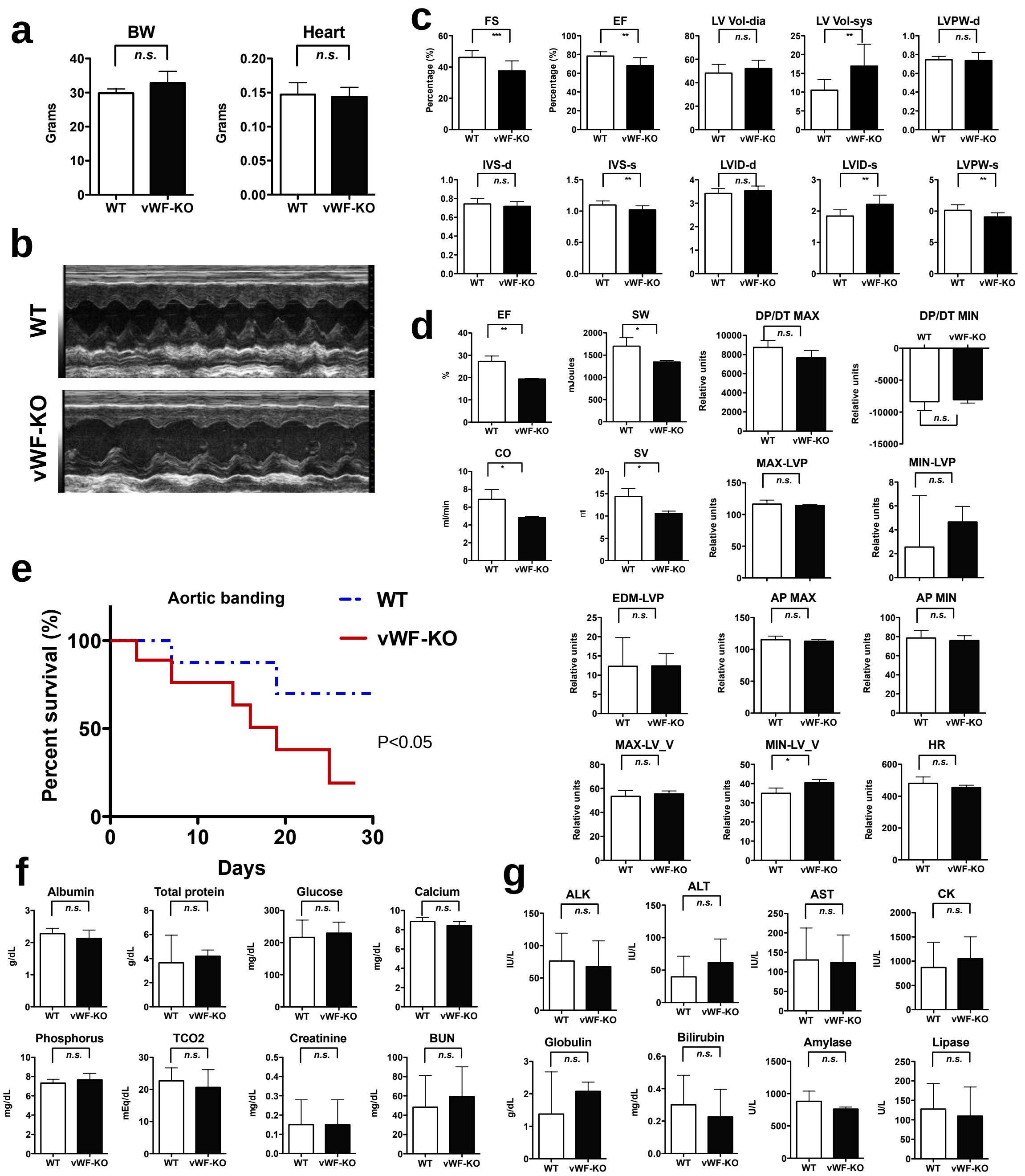

Supplementary Figure 9. Absence of vWF is associated with impaired cardiac function but normal liver or kidney function. a. Whole body weight (BW) and heart weight from wild type (WT) and vWF knockout mice (vWF-KO) (*n* = 12). **b.** Representative images of echocardiography in WT and KO mice. **c.** Echocardiographic analysis of heart size and function (*n* = 12). **d.** LV pressure-volume loop assay of cardiac function in WT and KO mice (*n* = 3; EF: ejection fraction, FS: fractional shortening; CO: cardiac output; SV: stroke volume; MAX/MIN-LVP = maximal/minimal left ventricular pressure; MAX/MIN-LV_V = maximal/minimal left ventricular volume; HR = heart rate; AP-MAX/MIN = maximal/minimal atrial pressure; DP/DT MAX/MIN = maximal/minimal dP/dT ration; EDM-LVP = end of diastole left ventricular

pressure). **e.** Survival of WT vs. vWF KO mice subjected to aortic banding ($n = 8$ in each group). **f-g.** Sera were collected from vWF-KO or WT mice for chemical analysis of liver function (**f**) and renal function (**g**). ALK = alkaline phosphatase; ALT = alanine amino-transferase; AST = aspartate aminotransferase; BUN = blood urea nitrogen. Error bars: standard deviation. $n = 3$, with 3 replicates. Two-sided *t*-test was performed in all significance tests. *p<0.05; **p<0.01; ***p<0.001; ****p<0.0001; n.s. p>0.05.

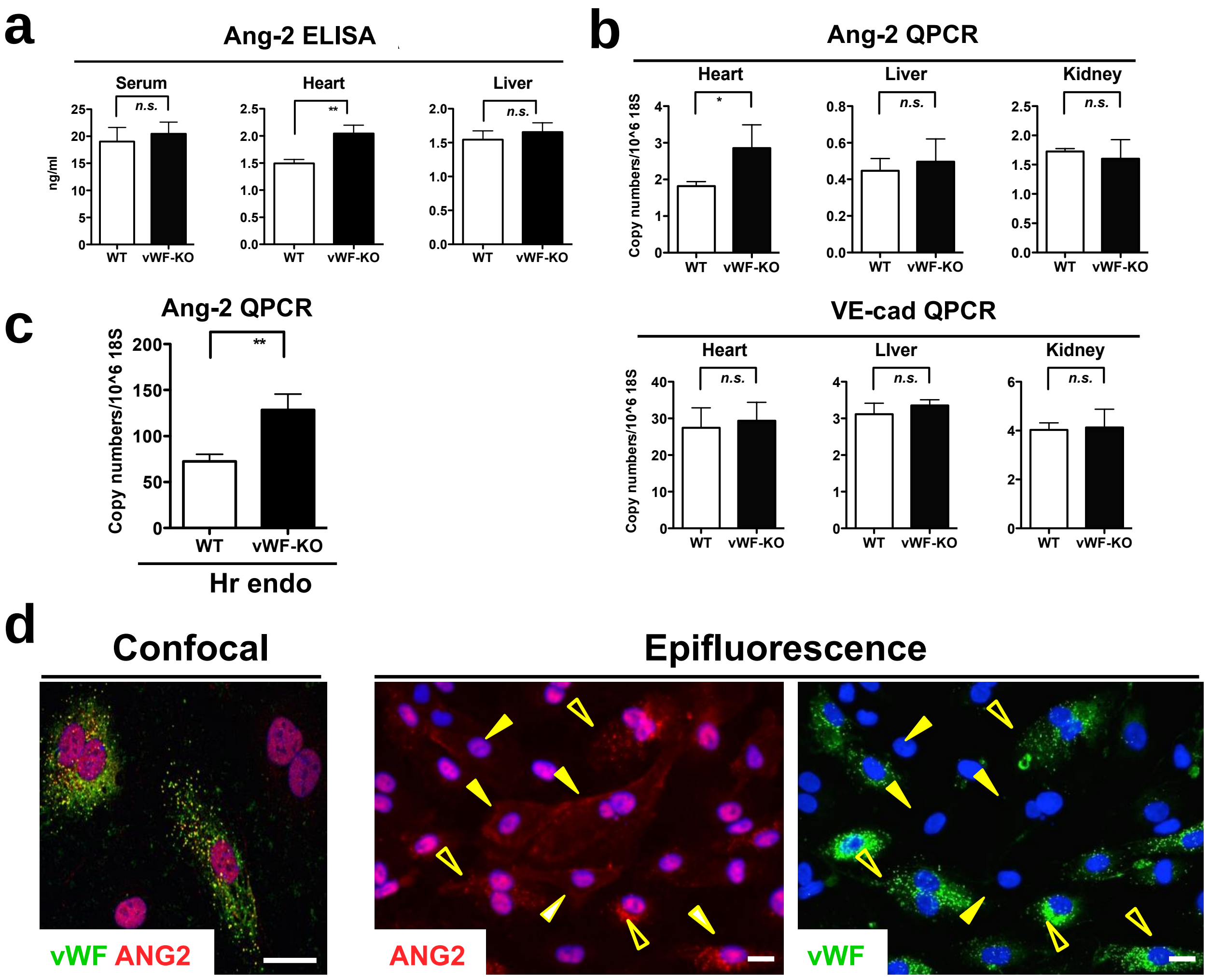

Supplementary Figure 10. Absence of vWF is associated with upregulation of Ang2 in the heart. a. Angiopoietin 2 (Ang2) protein levels in serum, heart and liver of WT and vWF KO mice (ELISA, n = 5). **b.** Ang2 (and VE-cadherin as a control) mRNA expression in whole heart, liver and kidney of WT and vWF KO mice (qPCR normalized to 18S RNA, represented as copy numbers per 10^6 18S; n = 3). **c.** Ang2 mRNA expression in cultured endothelial cells isolated from WT and vWF KO hearts (n = 3). **d.** Immunofluorescent co-staining for vWF and Ang2 protein in human heart microvascular endothelial cells (*left*, confocal; *right*, epifluorescence microscopy; *filled arrows*, vWF-negative cells with a pronounced cytoplasmic Ang2 blush; *empty arrows*, vWF-positive cells with punctate co-staining of vWF and Ang2, colocalized in WPBs). Sclae bar: 20µm.

SUPPLEMENTARY TABLES

Supplementary Table 1: List of primer sequences

NAME	PRIMER SEQUENCES
**QPCR**	
mVWF-F	5’- TGGATCCCGAGTCCTTTGTG-3’
mVWF-R	5’-GAGTACAGGACATGCGCACTCT-3’
hVWF-F	GTCGAGCTGCACAGTGACATG
hVWF-R	GCACCATAAACGTTGACTTCCA
**Bisulfite sequencing**	
hVWF-upstream-outer-F	5’-AGT TTT AGG GTA GGA GGT ATT ATG G-3’
hVWF-upstream-outer-R	5’-AAA TCA CAA TAA AAC AAC ACA ACT T-3’
hVWF-upstream-inner-F	5’-GAG GTA TTA TGG AGA AGT AGT GAG G-3’
hVWF-upstream-inner-R	R 5’-TAA TCC AAC TTC CAA AAC TAA ACT C-3’
hVWF-core-outer-F	5’-GGA GGT TAA TTT TTT GTT GTG GTA GT-3’
hVWF-core-outer-R	5’-TCT CCA AAC AAA TAT CCC CTC CCA ATC-3’
hVWF-core-inner-F	5’-GGG TGG TTG GTG GAT GTT AT-3’
hVWF-core-inner-R	5’- AAA CAA ACT TCA TTC CAA CTC ATT TA-3’
mVWF-prox-outer-F	5’-GTT TTT GTT TTT AGG TAT GT-3’
mVWF-prox-outer-R	5’-TCA AAT TAT CCA AAA ACT TC-3’
mVWF-prox-inner-F	5’- GGT TAG GTT TTT GTG AGG TT-3’
mVWF-prox-inner-R	5’-CCT CTA CCT ACA ATA ACT CC-3’
mVWF-exon-outer-F	5’- ATT TGG AGT TAT TGT AGG TAG A-3’
mVWF-exon-outer-R	5’-AAC CAT AAA ATC CAA ATA ACT T-3’
mVWF-exon-inner-F	5’-GAT TTT GAA GTT TTT GGA TA-3’
mVWF-exon-inner-R	5’-CTC CAA ACA AAT CCC TTC TA-3’
**Pol II ChIP**	
hVWF -829/-780 F	5’-TGC CTC AGG GTG CTT GTC TC-3’
hVWF -829/-780 R	5’-GAT GAC TCT CGT GCT GGG ATT C-3’
hVWF -480/-380 F	5’-GCC GAT CCA TTC AAC CCT G-3’
hVWF -480/-380 R	5’-GGA GTA CTA ACA CTT CCT ATG CAC CC-3’
hVWF 124/233 F	5’-CTG TAG CAG ACC TGA TTG AGC C-3’
hVWF 124/233 R	5’-GGC TGC GGC TAT CTC CAA G- 3’
**ChIP-bisulfite sequencing**	
hVWF-upstream-ChIP-outer-F	5’-TTG GAA TTT AGT GGT TTT TTA-3’
hVWF-upstream-ChIP-outer-R	5’-ACA TTT CCA CCC ACT TCT-3’
hVWF-upstream-ChIP-inner-F	5’-GAG GAG GTT TTT TAT ATT T-3’
hVWF-upstream-ChIP-inner-R	5’-ACC CAC TTC TAA TAC CCT A-3’
hVWF-core-outer-F	5’-TTG TGG TGG GAA AGG GA-3’

hVWF-core-outer-R	5'-CCC CAA AAC CCT CAA AA-3'
hVWF-core-inner-F	5'-AGG GTG GTT GGT GGA TGT-3'
hVWF-core-inner-R	5'-AAA ATA TTA AAA TCA TCC CTA-3'
**Hairpin bisulfite sequencing**	
hVWF-hairpin-F	5'-TTTTTTAGGAGTAGGGATTA-3'
hVWF-hairpin-R	5'- CTCCCCAAAAACAAAAATCA-3'
hVWF-hairpin-nested--F	5'-ATTAGTTAGTTTTGTATTTTTT-3'
hVWF-hairpin-nested--R	5'-ATCAATCCTACATCTTCCTCCC-3'

Supplementary Table 2: Parameters of the two-Gaussian mixture fits to vWF mRNA distributions in cultured ECs.

EC type	w_{Low}	w_{High}	σ_{Low}	σ_{High}	AIC
HUVEC	0.07	0.93	0.05	0.34	100
HPAEC	0.85	0.15	0.11	0.27	-68
HCAEC	0.22	0.78	0.05	0.34	85
HAoEC	0.3	0.7	0.08	0.32	178
HMVEC-D	0.37	0.63	0.09	0.48	89
HMVEC, 1st prep	0.38	0.62	0.08	0.5	66
HMVEC, 2nd prep	0.43	0.57	0.1	0.27	83

Supplementary Table 3: Parameters of the two-Gaussian mixture fits to vWF mRNA distributions in clonally derived EC populations.

EC clone	w_{Low}	w_{High}	σ_{Low}	σ_{High}	AIC
HUVEC Clone 1	0.14	0.86	0.09	0.34	138
HUVEC Clone 2	0.09	0.91	0.05	0.41	288
HAoEC Clone 1	0.52	0.48	0.1	0.18	18
HAoEC Clone 2	0.35	0.65	0.12	0.26	146
HPAEC Clone 1	0.59	0.41	0.09	0.38	68
HPAEC Clone 2	0.74	0.26	0.07	0.42	64
HPAEC Clone 3	0.89	0.11	0.12	0.37	-52

SUPPLEMENTARY NOTES

SUPPLEMENTARY NOTE 1. Stochastic OFF→ON transition-driven population dynamics and transition rate measurement. To understand time-dependent accumulation of LacZ in vWF-Cre-ROSA26R endothelial cells (ECs), we created a population dynamics model driven by four simple assumptions: *i)* transitions between ON and OFF promoter activity states are stochastic, with random rates P_{ON} and P_{OFF}; *ii)* transition rates are independent of cell division; *iii)* single-cell level transitions do not depend on the state of neighboring ECs; and *iv)* LacZ reporter levels in single ECs are well characterized by a binary ON/OFF value. Using the above assumptions, we calculated the time-dependent fraction of LacZ-negative vWF-Cre-ROSA26R ECs subject to random transitions from OFF to ON state, as well as expansion/shrinkage of the EC population due to proliferation/cell loss. The total number of ECs, $N(t)$, thus expands as:

$$N(t) = N(0) \cdot \exp\{ \int_0^t [P_D(t) - P_L(t)]\, dt\}. \quad (1)$$

The number of LacZ-negative ECs is increased by cell division, and decreased by cell loss and random OFF-to-ON transitions, according to:

$$dN_{OFF}(t)/d\underline{t} = [P_D(t) - P_L(t)]\cdot N_{OFF}(t) - P_{ON}\cdot N_{OFF}(t) \Rightarrow \quad (2)$$

$$N_{OFF}(t) = N_{OFF}(0) \cdot \exp\{ \int_0^t [P_D(t) - P_L(t)]\, dt\} \cdot \exp(- P_{ON}\cdot t). \quad (3)$$

The time-dependent fraction of LacZ-negative ECs is thus given by:

$$N_{OFF}(t)/N(t) = N_{OFF}(0)/N(0) \cdot \exp(- P_{ON}\cdot t). \quad (4)$$

Consequently, this simple model predicts that the LacZ-negative population decreases exponentially with time. To verify this, we measured the fraction of cells in the ON state by counting LacZ/CD31 double positive ECs in heart and liver tissue sections from vWF-Cre-ROSA26R mice, sacrificed on days 1, 4, 7, 21 and 28 after birth (**Fig. 3c-e)**. Linear regression on

the log-normal plot in **Figure 3e** (time after birth approximated as ~12h and 3.5, 6.7, 20.5, 27.5 days) leads to:

$$P^{Heart}_{ON} = 0.019 \pm 0.003 \text{ /day/EC} \quad (5)$$

$$P^{Liver}_{ON} = -\ 0.0001 \pm 0.0002 \text{ /day/EC} = 0. \quad (6)$$

(In estimating the standard deviations of slope and intercept we assumed normally distributed residual errors).

These results allowed us to estimate the percentage of LacZ negative cells *at birth*:

$$N^{Heart}_{OFF}(0)/N(0) = 10^{(1.85 \pm 0.05)}\ \% = 70 \pm 1\ \% \quad (7)$$

$$N^{Liver}_{OFF}(0)/N(0) = 10^{(1.974 \pm 0.003)}\ \% = 94 \pm 1\ \% \quad (8)$$

Assuming that P_{ON} does not change during embryonic development, the equation predicts the starting point of vWF mosaic expression in the heart: $N_{OFF}(t_{start})/N(0) = 100\ \%$ when $t_{start} = -8$ days before birth, around embryonic day E11.0.

Next, we assumed that the in adult vWF$^{LacZ/+}$ knock-in mice, the fraction of LacZ-positive/negative cells, f^{KI}_{ON} and f^{KI}_{OFF} have reached an equilibrium by day 28 (a balance between ON/OFF transitions). Thus:

$$P_{ON}{\cdot}N^{KI}_{OFF} = P_{OFF}{\cdot}N^{KI}_{ON} \quad \Rightarrow \quad P_{OFF} = P_{ON}{\cdot}\ f^{KI}_{OFF}/f^{KI}_{ON}. \quad (9)$$

Our measurements indicate that $f^{KI}_{ON} = 0.38 \pm 0.1$ in the heart, and 0.054 ± 0.04 in the liver. The OFF rates are thus:

$$P^{Heart}_{OFF} = 0.03\text{/day/EC, and } P^{Liver}_{OFF} = 0. \quad (10)$$

SUPPLEMENTARY NOTE 2. Gaussian mixture fitting of the vWF mRNA distribution in single ECs. The number of vWF and VE-cadherin (CDH5) mRNA molecules in single cells was

measured by FISH staining in 6 types of human ECs: human umbilical vein ECs (HUVEC), human pulmonary ECs (HPAEC), human coronary artery ECs (HCAEC), human aortic ECs (HAoEC), human dermal microvascular ECs (HMVEC-D) and two separate preparations of human cardiac microvascular ECs (HMVEC). The distribution of the vWF to VE-cadherin mRNA molecule ratios in single cells ($r = N_{\mathrm{vWF}}/N_{\mathrm{CDH5}}$) was fitted by a Gaussian mixture model with two normal subpopulations:

$$P(r) = w_{\mathrm{Low}}\cdot \mathrm{G}_{\mathrm{Low}}(r, \mu_{\mathrm{Low}}, \sigma_{\mathrm{Low}},) + w_{\mathrm{High}}\cdot \mathrm{G}_{\mathrm{High}}(r,\mu_{\mathrm{High}}, \sigma_{\mathrm{High}}), \qquad (11)$$

where $\mathrm{G}(r, \mu, \sigma) = \exp[- (r - \mu)^2/(2\sigma)] / (2\pi\sigma)^{0.5}$ represents the normal distribution. We used an Expectation-Maximization algorithm based on code from [1]. As the position of the two peaks, μ_{Low} and μ_{High} was very similar among the EC subtypes, we searched for a global pair of peaks that maximized the average Akaike information criterion (AIC) among the distribution fits for the 7 EC preparations: $\mu_{\mathrm{Low}} = 0.115$ and $\mu_{\mathrm{Low}} = 0.79$. We then identified the best fitting Gaussian mixture with fixed μ values, and determined the weights and standard deviations of the two subpopulations for each EC subtype. vWF-low and -high percentages on **Figures 4,5** and **Supplementary Figure 6b** represent the weights of the two fitted Gaussians (**Supplementary Table 2**).

In clonal ECs derived from HAoECs and HUVECs, the vWF-high population had slightly higher vWF to VE-cadherin mRNA ratios compared with their parent distributions. We thus re-optimized the value of μ_{High} by finding the best fit across 2 HAoEC and 2 HUVEC clonal populations, and used this new $\mu_{\mathrm{High}} = 0.95$ to fit all clones ($\mu_{\mathrm{Low}} = 0.115$, $\mu_{\mathrm{High}} = 0.95$; **Supplementary Table 3**).

SUPPLEMENTARY NOTE 3. Ruling out alternative mechanisms of mosaic heterogeneity among isogenic neighbors that are not supported by our data. In addition to biological noise toggling a bistable switch, additional mechanisms exist for generating phenotypic heterogeneity among isogenic cells. These include excitable circuits (monostable systems in which noise can trigger prolonged but transient "excursions" through state space) [2], cell-autonomous oscillatory circuits [3,4] and pattern-forming regulatory mechanisms (e.g., lateral inhibition) [5]. Both excitable and oscillatory circuits can give rise to dynamic phenotypic mosaics, but neither can generate static, locked-in mosaicism. Our LacZ staining data in the aortae of one year-old VWF-Cre-ROSA26R mice, however, unequivocally demonstrate that VWF expression is a static mosaic in this vascular bed (**Supplementary Fig. 3**). To rule out pattern-generating mechanisms leading to differences between neighboring cells, we compared the neighborhood of vWF ON vs. OFF cells *in vitro* (**Supplementary Figs. 6c-e**), and found no relationship between the vWF-state of an EC and its neighborhood. Taken together, our results appear incompatible with excitable, oscillatory or pattern-forming regulatory mechanisms, but are consistent with a bistable switch with organ-specific barrier and noise-sensitivity.

SUPPLEMENTARY NOTE 4. Estimating the sampling error of average DNA methylation from bisulfite sequencing. Bisulfite sequencing is limited to sampling n = 15-20 individual DNA alleles from a dish of ~10^6 cells. Consequently, the frequency at which an individual pattern of DNA methylation appears is measured with considerable sampling error. We sought to determine the magnitude of sampling error of measured average DNA methylation in a population, where each sequenced DNA allele has 4 CpG sites (correct for all sequenced human

vWF promoter fragments). To do this, we simulated the experiment of sampling a dish of ECs by random selection of n = 15 alleles. For each individual simulated dish, we assigned random weights w_i to all possible CpG methylation patterns (2^4 = 16 distinct ones in our case; w_i represents the fraction of DNA alleles in the dish with the ith CpG methylation pattern: $\sum_i w_i = 1$). Based on these weights, we computed the exact value of average DNA methylation across the 4 CpG sites in the *in silico* dish. To estimate the *error* with which a bisulfite sequencing experiment that covers n = 15 alleles measures this average, we conducted 5000 in silico bisulfite sequencing experiments (with 15 alleles each), and the average *error* of average DNA methylation. To test the dependence of this error on the homogeneity of the patterns in the dish, we repeated our error estimate for 400 distinct *in silico* dishes (with different distributions CpG patterns), and plotted the average error of average DNA methylation as a function of the entropy of the w_i weights, $H = - \sum_i w_i \cdot \ln(w_i)$ (e.g., i = 1 for ○○○○, i = 2 for ○○○●, and i = 16 for ●●●●; at low entropy, a few patterns largely dominate the distribution – low heterogeneity, while at high entropy all 16 patterns are relatively evenly distributed – high heterogeneity). As **Supplementary Figure 7a** indicates, for alleles with 4 CpG sites and 15 sequenced alleles, the error of average DNA methylation is ≃ 0.05, or around 5%. Horizontal error bars in **Figures 5b** and **6b** show a 5% standard error.

SUPPLEMENTARY NOTE 5. Estimating the likelihood that the DNA methylation pattern in clonal ECs is faithfully replicated from the original parent cell. Nearly all clonal EC populations we assayed harbored a variety of DNA methylation patterns, indicating less than perfect fidelity in the inheritance of the methylation pattern on the two parent cell alleles. In order to estimate the probability of observing these patterns due to experimental error (i.e., from

a population that *maintained* two patterns) we measured the average bisulfite conversion error (fraction of C nucleotides *not* in a CpG site and *not converted* to T during bisulfite conversion: $p_{\text{CONV}} = 0.0026 \pm 0.01$/nucleotide for the upstream region and $p_{\text{CONV}} = 0.0009 \pm 0.0009$/nucleotide for the exon 1 region) and T-to-C sequencing error (fraction of T nucleotides *incorrectly* sequenced as C on individual bisulfite-converted DNA alleles: $p_{\text{T->C}} = 0.0006 \pm 0.0004$/nucleotide for the upstream region and $p_{\text{T->C}} = 0.0008 \pm 0.0005$/nucleotide for the exon 1 region) across all our bisulfite sequencing experiments. Next, we generated 100,000 *in silico* experiments for each experimental bisulfite-sequencing series performed on a clonal dish, matching the number of "sequenced" DNA strands in each *in silico* set to the *in vitro* set. Each *in silico* DNA strand was assumed to harbor one of the two most prevalent methylation patterns seen in the corresponding *in vitro* set (e.g., oooo or ●●oo on **Fig. 7a**, HUVEC clone 1, upstream). This strand was then "converted and sequenced" *in silico* with the above errors. In the resulting mock ensemble, we calculated the likelihood of obtaining a pattern with as many mismatching CpG sites as seen *in vitro* (**Supplementary Fig. 7e**): we measured the likelihood that at least 1 or 2 *in vitro* CpG site mismatches appeared due to experimental error, and calculated the p-value p_i of observing n_i alleles with at least i mismatched CpGs (n_i is the in vitro number of stands with i mismatching CpGs compared to the two dominant patterns). In the result list below, regions that pass the non-clonality test marked in bold:

* PAEC Clone 1 - ***upstream***: $n_1 = 4, n_2 = 0$ => $\boldsymbol{p_1 < 10^{-5}}$, $p_2 = 1$; ***exon 1***: $\boldsymbol{n_1 = 5, n_2 = 2, n_3 = 1}$ => $\boldsymbol{p_1 < 10^{-5}, p_2 < 10^{-5}, p_3 < 10^{-5}}$
* PAEC Clone 2 - ***upstream***: $n_1 = 2, n_2 = 0$ => $\boldsymbol{p_1 = 0.01}$, $p_2 = 1$; ***exon 1***: $\boldsymbol{n_1 = 6, n_2 = 1}$=> $\boldsymbol{p_1 < 10^{-5}}$, $\boldsymbol{p_2 = 0.00012}$
* PAEC Clone 3 - ***upstream***: $n_1 = 3, n_2 = 0$ => $\boldsymbol{p_1 = 0.00039}$, $p_2 = 1$; ***exon 1***: $\boldsymbol{n_1 = 2}$, $n_2 = 0$ => $\boldsymbol{p_1 =}$

0.0045, $p_2 = 1$

* HAoEC Clone 1 - *upstream*: $n_1 = 1$; $n_2 = 0$ => $p_1 = 0.14$; $p_2 = 1$; ***exon 1***: $\boldsymbol{n_1 = 9}$, $\boldsymbol{n_2 = 2}$ => $\boldsymbol{p_1 < 10^{-5}}$, $\boldsymbol{p_2 < 10^{-5}}$

* HAoEC Clone 2 - *upstream*: $n_1 = n_2 = 0$ => $p_1 = p_2 = 1$; ***exon 1***: $\boldsymbol{n_1 = 3}$, $n_2 = 0$ => $\boldsymbol{p_1 = 0.00012}$, $p_2 = 1$

* HAoEC Clone 3 - *upstream*: $n_1 = n_2 = 0$ => $p_1 = p_2 = 1$; ***exon 1***: $\boldsymbol{n_1 = 5}$, $n_2 = 0$ => $\boldsymbol{p_1 < 10^{-5}}$, $p_2 = 1$

* HAoEC Clone 4 - *upstream*: $n_1 = n_2 = 0$ => $p_1 = p_2 = 1$; ***exon 1***: $\boldsymbol{n_1 = 2}$, $n_2 = 0$ => $\boldsymbol{p_1 = 0.0036}$, $p_2 = 1$

* HUVEC Clone 1 - ***upstream***: $\boldsymbol{n_1 = 5}$, $n_2 = 0$ => $\boldsymbol{p_1 < 10^{-5}}$, $p_2 = 1$

* HUVEC Clone 2 - ***upstream***: $\boldsymbol{n_1 = 6}$, $n_2 = 0$ => $\boldsymbol{p_1 < 10^{-5}}$, $p_2 = 1$

* HUVEC Clone 3 - ***upstream***: $\boldsymbol{n_1 = 2}$, $n_2 = 0$ => $\boldsymbol{p_1 = 0.011}$, $p_2 = 1$

- HUVEC Clone 4 - *upstream*: $n_1 = n_2 = 0$ => $p_1 = 0.13$, $p_2 = 1$

- HUVEC Clone 5 - *upstream*: $n_1 = n_2 = 0$ => $p_1 = 1$, $p_2 = 1$

- HUVEC Clone 6 - *upstream*: $n_1 = 1$, $n_2 = 0$ => $p_1 = 0.14$, $p_2 = 1$

In summary, we have found that sequencing series in which at least 2 DNA strands deviate from the two dominant patterns are statistically unlikely to arise due to experimental error (i.e., from clonal populations in terms of DNA methylation). Specifically, DNA methylation is ***not*** faithfully maintained in the exon 1 region of any of the 3 HPAEC or 4 HAoEC clones. Moreover, in 3 of 6 HUVEC clones where the parent exon 1 region is almost entirely unmethylated, the heterogeneity of the upstream segment is nonetheless highly unlikely be an artifact of conversion and/or sequencing.

SUPPLEMENTARY NOTE 6. Estimating methylation maintenance, loss and de novo methylation from hairpin bisulfite sequencing. In 1990 Pfeifer *et al* published an influential mathematical model of dynamic DNA methylation, used to put a lower bound on the maintenance rate of DNA methylation at a CpG island on an X-linked genetic locus [6]. The subsequent development of hairpin bisulfite sequencing allows for a direct estimate of this rate, along with that of *de novo* methylation (relative to maintenance). This method assumes that a) fully methylated or fully unmethylated CpG/CpG dyads reflect accurate transmission of methylated or unmethylated cytosine states during the preceding DNA replication; and b) hemimethylated dyads represent either a failure to maintain epigenetic fidelity at/after the last replication event, or *de novo* methylation in progress. Thus, the fidelity of maintaining methylation is measured by assigning the top strand as parent (the strand vWF is read off of), measuring the ratio of *symmetrically* methylated vs. methylated CpGs, then repeating this process with the bottom strand designated as parent. This yields two similar, but non-identical estimates of DNA methylation fidelity, a minimum and maximum bound. As the true parent and daughter strands are equally likely to be sampled via hairpin bisulfite sequencing, the average of these two is a good approximation of E_m at the locus of interest. The fidelity of maintaining un-methylated CpGs, E_u, can be similarly estimated as the ratio of *symmetrically* un-methylated vs. un-methylated CpGs on the top, then bottom strands. E_u can be used to calculate the rate of de novo methylation as $E_d = 1 - E_u$.

* <u>HUVEC</u>: E_m cannot be estimated at the core promoter, since no methylation is observed. The data indicates that $E_u = 1$ and $E_d = 0$, satisfying the $M = E_d/(1+E_d - E_m) = 0$ equilibrium condition for any $E_m \in [0, 1]$.

* HMVEC:

- MM = 1 symmetrically methylated CpG dyad;

- M_{top} = 5 => E_m (t) = 0.2; M_{bottom} = 4 => E_m (b) = 0.25; **$\langle E_m \rangle$ = 0.225**;

- UU = 25 symmetrically un-methylated CpG dyads;

- U_{top} = 28 => E_u (t) = 0.89; U_{bottom} = 29 => E_u (b) = 0.86; **$\langle E_u \rangle$ = 0.877**;

=> **$\langle E_d \rangle$ = 0.123** ($E_d \in [0.11, 0.14]$).

* HPAEC:

- MM = 2 symmetrically methylated CpG dyads;

- M_{top} = 6 => E_m (t) = 0.33; M_{bottom} = 4 => E_m (b) = 0.5; **$\langle E_m \rangle$ = 0.42**;

- UU = 22 symmetrically un-methylated CpG dyads;

- U_{top} = 24 => E_u (t) = 0.92; U_{bottom} = 26 => E_u (b) = 0.85; **$\langle E_u \rangle$ = 0.881**;

=> **$\langle E_d \rangle$ = 0.119** ($E_d \in [0.08, 0.15]$).

* HCVSMC:

- MM = 16 symmetrically methylated CpG dyads;

- M_{top} = 19 => E_m (t) = 0.84; M_{bottom} = 16 => E_m (b) = 1; **$\langle E_m \rangle$ = 0.921**;

- UU = 8 symmetrically un-methylated CpG dyads;

- U_{top} = 8 => E_u (t) = 1; U_{bottom} = 11 => E_u (b) = 0.73; **$\langle E_u \rangle$ = 0.864**;

=> **$\langle E_d \rangle$ = 0.136** ($E_d \in [0, 0.27]$).

It is interesting to note that the only cell line for which the rate of methylation maintenance at the vWF locus comes close to the rates reported for tightly repressed CpG islands are vascular SMCs, in which vWF is completely off (E_m = 0.92). In contrast, ECs with medium average DNA methylation at the 3 assayed CpG sites have DNA maintenance rates below 50% per CpG per

division ($E_m \simeq 0.23$ in HMVEC and $E_m = 0.42$ in HPAEC). Rates of *de novo* methylation, on the other hand, are all within the relatively narrow range of 0.12 (HPAEC) and 0.14 (HCVSMC), regardless of cell type.

Our results on mosaic vWF expression and differential DNA methylation in ON vs. OFF cells points to a more complex DNA methylation/demethylation dynamics than the one observed on stably methylated or unmethylated CpG islands [6,7]. Instead of the dynamic equilibrium between steady loss and de novo methylation occurring at CpG islands with relatively constant average methylation, the vWF locus toggles back and forth between 2 alternate states. In the transcriptionally repressive OFF state, maintenance is high (though likely not as high as in SMCs), shifting the balance towards a methylated state. In the transcriptionally permissive ON state, however, either maintenance or de novo methylation or both are low, shifting the locus towards a completely unmethylated state. Due to stochastic flips between these two states, the populations we assayed with hairpin bisulfite are mixtures of these two. Consequently, our rate estimates for HMVEC and HPAEC are long-term weighted averages of these two sets of rates, occurring at the transcriptionally active vs. repressed promoter.

SUPPLEMENTARY NOTE 7. Bistability of promoter epigenetic states. Our results indicate that promoter DNA methylation is necessary for tight silencing of the vWF promoter (as evidenced by increased expression in both ECs and non-ECs in the presence of 5-AZA-induced reduction of DNA methylation), and sufficient to block vWF transcription (as evidenced by expression from methylated vs. unmethylated vWF promoter-reporter constructs). These results

raise the question of whether DNA methylation status of the vWF promoter could itself be the noise-sensitive bistable switch.

An elegant series of proof-of principle experiment by Hathaway *et al* have shown that repressive/permissive histone and heterochromatin formation alone can create bistability at the level of a single promoter, albeit with a relatively low barrier [8]. To show this, they silenced Oct4 in embryonic stem (ES) cells by tethering HP1 to its promoter. This tethered HP1 recruits repressive histone marks and packages the *Oct4* locus into heterochromatin, in spite of ES-specific factors that normally drive Oct4 transcription. At the single cell level, transcriptional silencing occurs stochastically, in an all-or-none fashion, over several days. Removal of tethered HP1 is sufficient for Oct4 re-expression, but this transition is also all-or-none, and far from immediate. Four days after HP1 tethering was removed, the *Oct4* locus remained silent in ~50% of ES cells, with 37% still negative at day 6. Overall, these results indicate that heterochromatin formation alone forms a barrier to transcription, albeit one that strong transcriptional activators can breach. Interestingly, DNA methylation / demethylation of this promoter can significantly increase the barrier between active and silenced promoter states. The increased barrier from a transcriptionally active state to a DNA methylated silent state is evidenced by very slow recruitment of DNA methylation, which only occurs with prolonged HP1 tethering and saturates after 4.5 weeks. Subsequent removal of HP1 tethering, ~70% of the cells were still maintaining Oct4 repression 6 days later, and 64% of clonally derived colonies were completely negative for Oct4 transcriptions, indicating the heritable maintenance of a silenced state. In the presence of activating transcription factors, however, cells are subject to slow stochastic transitions to an ultimately more stable active state.

The above described bistability results from interactions between DNA methylation, cooperative histone modifications and Pol-II mediated transcription, interactions that contain an essential double-negative feedback. To understand the role of this feedback, let us assume that DNA methylation of individual CpG sites spontaneously toggles ON/ OFF on our promoter of interest, with rates determined by the constant balance of activity between DNA methyltransferases and demethylating enzymes [9,10]. On a promoter with multiple CpG sites, this process alone creates a dynamically changing mixture of methylated and unmethylated CpGs, a mixture that fluctuates around an equilibrium number of methylated sites on each allele. This noisy process in itself is not sufficient to create bistability, as there is no barrier between transcriptionally permissive (e.g., completely unmethylated) and inhibitory (methylated) states. However, it is well established that DNA methylation of a promoter and Pol II activity are linked. ***First***, DNA methylation is known to block Pol II activity, as methylated CpGs are bound by proteins that promote repressive histone modifications (e.g., H3K9 di- and tri-methylation), which, in turn, directly inhibit Pol II [11-13]. ***Second***, active Pol II-mediated transcription blocks *de novo* DNA methylation, which is thought to occur only after permissive histone marks (especially H3K4me2-H3K4me3 82) are removed [8,14,15]. In a cellular environment where DNA methyltransferases and demethylating enzymes are balanced and active, and where activating transcription factors are present but not overwhelming, the above double-negative feedback could give rise to a dynamically toggling bistable switch. In this case, however, the stochastic binding/unbinding driving the switch's transitions would be *uncorrelated* between alleles. Our experiments rule out this type of promoter-intrinsic noise as the driver of vWF mosaic heterogeneity, and point, instead, to stochastic flips in an upstream signal that impacts both

alleles in concert (**Fig. 7c**).

Supplementary Note 8. vWF expression is required for cardiac health. One-micron Giemsa-stained sections of the heart from 14-week old (but not 2-week old) vWF-null mice demonstrated collapse of many capillaries in the right and left ventricles (**Supplementary Fig**. **8a-b** show left ventricle) and patches of frothy-appearing cardiomyocytes (**Supplementary Fig**. **8c-d**). Electron microscopy revealed profound focal ultrastructural abnormalities in the left and right ventricles of the vWF knockout mouse (**Supplementary Figs. 8e-h**). In these areas, capillary ECs were swollen and electron-lucent. However, the lateral borders were intact, the nuclei appeared normal and the cytoplasm contained many polyribosomes, arguing against a preparation artifact. Blebbing was observed from the surface of ECs and the lumen was often packed with membrane bound vesicles. In neighboring cardiomyocytes, many of the mitochondria were swollen and electron-lucent. By contrast, the ultrastructure of the aorta (where there is normally a fixed mosaic of vWF expression), the kidney and liver (where vWF is not normally expressed in the glomerulus and hepatic sinusoids, respectively) was normal (**Supplementary Figs. 8i-n**). However, the heart weight was similar in vWF-null compared with wild type mice (**Supplementary Fig. 9a** shows body and heart weight). To determine whether these ultrastructural abnormalities were associated with functional changes, we carried out echocardiography (**Supplementary Fig. 9b, 9c**) and pressure-volume loop (**Supplementary Fig. 9d**) experiments. These studies demonstrated a significant increase in left ventricle systolic volume (LV Vol-sys) and a reduction in fractional shortening (FS), ejection fraction (EF), stroke volume (SV) and cardiac output (CO) in vWF-null mice compared with wild type littermates, suggesting a systolic cardiac dysfunction. To stress the heart, we subjected mice to transverse

aortic constriction (**Supplementary Fig. 9e**). While perioperative mortality did not differ between genotypes, 30-day survival was significantly reduced in the vWF-null mice (**Supplementary Fig. 9e**). In contrast to these cardiac readouts, functional assays for liver and kidney were unchanged in vWF knockout mice (**Supplementary Figs. 9f-g**). Together, these findings indicate that vWF expression is necessary for cardiac health. They raise the possibility, but do not prove, that dynamic mosaicism in heart capillaries is responsible for this effect.

Supplementary Note 9. Ang2 is upregulated in vWF-negative endothelial cells. To determine whether loss of vWF may affect the storage and secretion of Ang2 in our model, we employed ELISA to measure Ang2 protein levels in our mice. Compared with wild type mice, vWF-null mice demonstrated significantly elevated levels of Ang2 levels in the heart, but not the liver or kidney (**Supplementary Fig. 10a**). Moreover, Ang2 levels were unchanged in the blood, suggesting that any pathological effect of Ang2 in the heart is related to local production in that organ. Interestingly, Ang2 mRNA expression was also selectively increased in the heart of vWF-null mice and in cultured cardiac microvascular ECs from these animals (**Supplementary Figs. 10b, 10c**). In cultured human cardiac MVEC, Ang2 co-localized with vWF in a subset of vWF-positive granules (consistent with WPB) (**Supplementary Fig. 10d**, left) [16]. In cells that lacked vWF, Ang2 staining appeared as a cytoplasmic “blush”, suggesting that the vWF OFF state is associated with Ang2 release through a secretory pathway (**Supplementary Fig. 10d**, right). Based on these data, we hypothesize that dynamic mosaicism of vWF expression in the capillaries of the heart is associated with randomly shifting hot spots of sustained Ang2 secretion, and that disruption of the mosaic leads to dysregulated Ang2 release and secondary microvascular damage.

SUPPLEMENTARY REFERENCES

1. Hao, J. *et al.* Precision Measurements of the Cluster Red Sequence Using an Error-Corrected Gaussian Mixture Model. *Ap J* **702,** 745–758 (2009).
2. Süel, G. M., Kulkarni, R. P., Dworkin, J., Garcia-Ojalvo, J. & Elowitz, M. B. Tunability and noise dependence in differentiation dynamics. *Science* **315,** 1716–1719 (2007).
3. Loewer, A. & Lahav, G. We are all individuals: causes and consequences of non-genetic heterogeneity in mammalian cells. *Curr Opin Genet Dev* **21,** 753–758 (2011).
4. Nelson, D. E. *et al.* Oscillations in NF-kappaB signaling control the dynamics of gene expression. *Science* **306,** 704–708 (2004).
5. Blanco, R. & Gerhardt, H. VEGF and Notch in tip and stalk cell selection. *Cold Spring Harb Perspect Med* **3,** a006569–a006569 (2013).
6. Pfeifer, G. P., Steigerwald, S. D., Hansen, R. S., Gartler, S. M. & Riggs, A. D. Polymerase chain reaction-aided genomic sequencing of an X chromosome-linked CpG island: methylation patterns suggest clonal inheritance, CpG site autonomy, and an explanation of activity state stability. *Proc Natl Acad Sci U S A* **87,** 8252–8256 (1990).
7. Laird, C. D. *et al.* Hairpin-bisulfite PCR: assessing epigenetic methylation patterns on complementary strands of individual DNA molecules. *Proc Natl Acad Sci U S A* **101,** 204–209 (2004).
8. Hathaway, N. A. *et al.* Dynamics and memory of heterochromatin in living cells. *Cell* **149,** 1447–1460 (2012).
9. Métivier, R. *et al.* Cyclical DNA methylation of a transcriptionally active promoter. *Nature* **452,** 45–50 (2008).
10. Jurkowska, R. Z., Jurkowski, T. P. & Jeltsch, A. Structure and Function of Mammalian DNA Methyltransferases. *Chem BioChem* **12,** 206–222 (2010).
11. Nan, X. *et al.* Transcriptional repression by the methyl-CpG-binding protein MeCP2 involves a histone deacetylase complex. *Nature* **393,** 386–389 (1998).
12. Fuks, F. *et al.* The methyl-CpG-binding protein MeCP2 links DNA methylation to histone methylation. *J Biol Chem* **278,** 4035–4040 (2003).
13. Ng, H. H., Jeppesen, P. & Bird, A. Active repression of methylated genes by the chromosomal protein MBD1. *Mol Cell Biol* **20,** 1394–1406 (2000).
14. Cedar, H. & Bergman, Y. Linking DNA methylation and histone modification: patterns and paradigms. *Nat Rev Genet* **10,** 295–304 (2009).
15. Jones, P. A. P. Functions of DNA methylation: islands, start sites, gene bodies and beyond. *Nat Rev Genet* **13,** 484–492 (2012).
16. Fiedler, U. *et al.* The Tie-2 ligand angiopoietin-2 is stored in and rapidly released upon stimulation from endothelial cell Weibel-Palade bodies. *Blood* **103,** 4150–4156 (2004).
